# Characterization of the Transcriptome of the Xerophyte *Ammopiptanthus mongolicus* Leaves under Drought Stress by 454 Pyrosequencing

**DOI:** 10.1371/journal.pone.0136495

**Published:** 2015-08-27

**Authors:** Tao Pang, Lili Guo, Donghwan Shim, Nathaniel Cannon, Sha Tang, Jinhuan Chen, Xinli Xia, Weilun Yin, John E. Carlson

**Affiliations:** 1 National Engineering Laboratory for Tree Breeding, College of Biological Sciences and Technology, Key Laboratory for Silviculture and Conservation, Beijing Forestry University, Beijing, People’s Republic of China; 2 College of Agricultural, Henan University of Science and Technology, Luoyang, People’s Republic of China; 3 The Schatz Center for Tree Molecular Genetics, Department Ecosystem Science and Management, Pennsylvania State University, University Park, Pennsylvania, United States of America; 4 Department of Forest Genetic Resources, Korea Forest Research Institute, Suwon 441–350, Korea; National Institute of Plant Genome Research, INDIA

## Abstract

**Background:**

*Ammopiptanthus mongolicus* (Maxim. Ex Kom.) Cheng f., an endangered ancient legume species, endemic to the Gobi desert in north-western China. As the only evergreen broadleaf shrub in this area, *A*. *mongolicus* plays an important role in the region’s ecological-environmental stability. Despite the strong potential of *A*. *mongolicus* in providing new insights on drought tolerance, sequence information on the species in public databases remains scarce. To both learn about the role of gene expression in drought stress tolerance in *A*. *mongolicus* and to expand genomic resources for the species, transcriptome sequencing of stress-treated *A*. *mongolicus* plants was performed.

**Results:**

Using 454 pyrosequencing technology, 8,480 and 7,474 contigs were generated after *de novo* assembly of RNA sequences from leaves of untreated and drought-treated plants, respectively. After clustering using TGICL and CAP3 programs, a combined assembly of all reads produced a total of 11,357 putative unique transcripts (PUTs). Functional annotation and classification of the transcripts were conducted by aligning the 11,357 PUTs against the public protein databases and nucleotide database (Nt). Between control and drought-treated plants, 1,620 differentially expressed genes (DEGs) were identified, of which 1,106 were up-regulated and 514 were down-regulated. The differential expression of twenty candidate genes in metabolic pathways and transcription factors families related to stress-response were confirmed by quantitative real-time PCR. Representatives of several large gene families, such as WRKY and P5CS, were identified and verified in *A*. *mongolicus* for the first time.

**Conclusions:**

The additional transcriptome resources, gene expression profiles, functional annotations, and candidate genes provide a more comprehensive understanding of the stress response pathways in xeric-adapted plant species such as *A*. *mongolicus*.

## Introduction

Drought stress prevents plants from realizing their full genetic potential, and greatly inhibits plant growth and productivity in agriculture in arid and semiarid areas [1]. By disrupting water balance, drought stress reduces plant development, reproduction, seed, and fruit yields [2]. Plants respond to drought stress with complex cascades of gene expression and signal transduction [3]. The identification and application of genes associated with stress tolerance are of interest in the development of drought tolerance in crop plants [4]. Approximately 2000 drought-responsive genes have been identified in *Arabidopsis thaliana* [5]. For some xerophytes, like *Populus euphratica*, gene discovery and transcriptome analyses have led to the identification of numerous potential stress-related genes [6–8]. These studies are contributing new gene resources, revealing drought responsive mechanisms in xerophytes and enabling production of more stress tolerant plants.

*Ammopiptanthus mongolicus* (Maxim. Ex Kom.) Cheng f., is a relatively primitive species found in the semi-arid region of north-western China [9]. The genus *Ammopiptanthus* (in the *Leguminosae*) contains only two species (*A*. *mongolicus* and *A*. *nanus*) which are considered endangered. This is of great concern ecologically given that the two species are the only evergreen broadleaf shrubs in this largely desert area [10]. Strong osmotic stress resistance makes it possible for *A*. *mongolicus* to survive in very inhospitable areas, where the annual precipitation can be as low as 150 mm and the lowest temperature is below -30°C. Our previous studies have elucidated cold resistance mechanisms in *A*. *mongolicus*. It is of even greater importance to discover the genes and metabolic pathways associated with drought stress tolerance in *A*. *mongolicus* [11]. Individual drought-related genes have been cloned and identified, such as *AmCBL1* [12], *AmNHX2* [13], *AmVP1* [14], *AmEBP1* [15] and *AmLEA* [16]. However, the few gene resources for *A*. *mongolicus* in GenBank (5,801 ESTs and 164 nucleotide sequences as of Dec 1^st^, 2014) are inadequate to fully characterize stress response pathways.

Originally, Sanger sequencing of cDNA libraries, which is relatively low throughput, costly and nonquantitative, was used for gene discovery and transcriptome analysis [17]. In recent years, rapid developments in next-generation sequencing (NGS), such as massively parallel 454 pyrosequencing and Illumina-based RNASeq, have spurred rapid developments in genomics and transcriptomics research [18]. Because of its advantages of longer read lengths, speed and accuracy, 454 pyrosequencing has been widely applied in *de novo* sequencing, especially in gene discovery and functional identification with non-model organisms, such as *Aegilops variabilis* [19], *Metasequoia glyptostroboides* [20], *Cicer arietinum* [21], *Eucalyptus grandis* [22], *Phalaenopsis orchids* [23], *Populus euphratica* [24], and *Liriodendron tulipifera* [25, 26].

A few studies have reported gene expression profiles from *A*. *mongolicus* under drought stress. Guo *et al*. analysed differential gene expression under cold and drought stress using complementary DNA-amplified fragment length polymorphism (cDNA-AFLP) technology [27]. They identified several drought-responsive genes associated with transcription, metabolic processes and protein modification. Zhou *et al*. reported the identification of candidate genes related to drought stress from the *A*. *mongolicus* root transcriptome using 454 pyrosequencing technology [28]. Also, Liu *et al*. [29] reported 5,282 ESTs from a cDNA library prepared from *A*. *mongolicus* plants under cold and drought stresses. Of the 1,594 putatively unique transcripts assembled from the 5,282 ESTs, 528 were specific to stress-response. Recently, Wu *et al*. [30] reported the transcriptome analysis of *A*. *mongolicus* using dehydration treatment on filter paper, identifying 2,028 DEGs in common across three time points (2, 8, 24 h).

Here, we describe the analysis of gene expression patterns in seedlings of the xerophyte *A*. *mongolicus* subjected to drought stress by extended water deprivation simulating natural drought conditions. Two cDNA libraries constructed from RNA of leaves from drought-treated seedlings and untreated seedlings were sequenced on a 454 pyrosequencing platform. Insights into the functions of expressed genes were obtained from COG annotations, GO classifications and KEGG metabolic pathway analysis. The putative functions of transcripts from leaves observed in this study represent a different set of genes from the previous reports from transcriptomes of root tissues and seedlings under drought and non-drought conditions. This report focuses on discovery of differentially expressed transcription factor genes and transcripts classified as ‘response to stimulus’, and their potential roles in regulating important stress-response pathways in *A*. *mongolicus*.

## Results

### Water potential of *A*. *mongolicus* subjected to drought stress

After four weeks exposure to drought stress conditions, the water potential of control samples (CK) and drought-treated samples (DT) from *A*. *mongolicus* plants measured in triplicate using the PSYPRO water potential system were -0.936 ± 0.040 Mpa and -1.618 ± 0.082 Mpa, respectively.

### 454 pyrosequencing read and *de novo* assembly metrics

**Fig 1:**
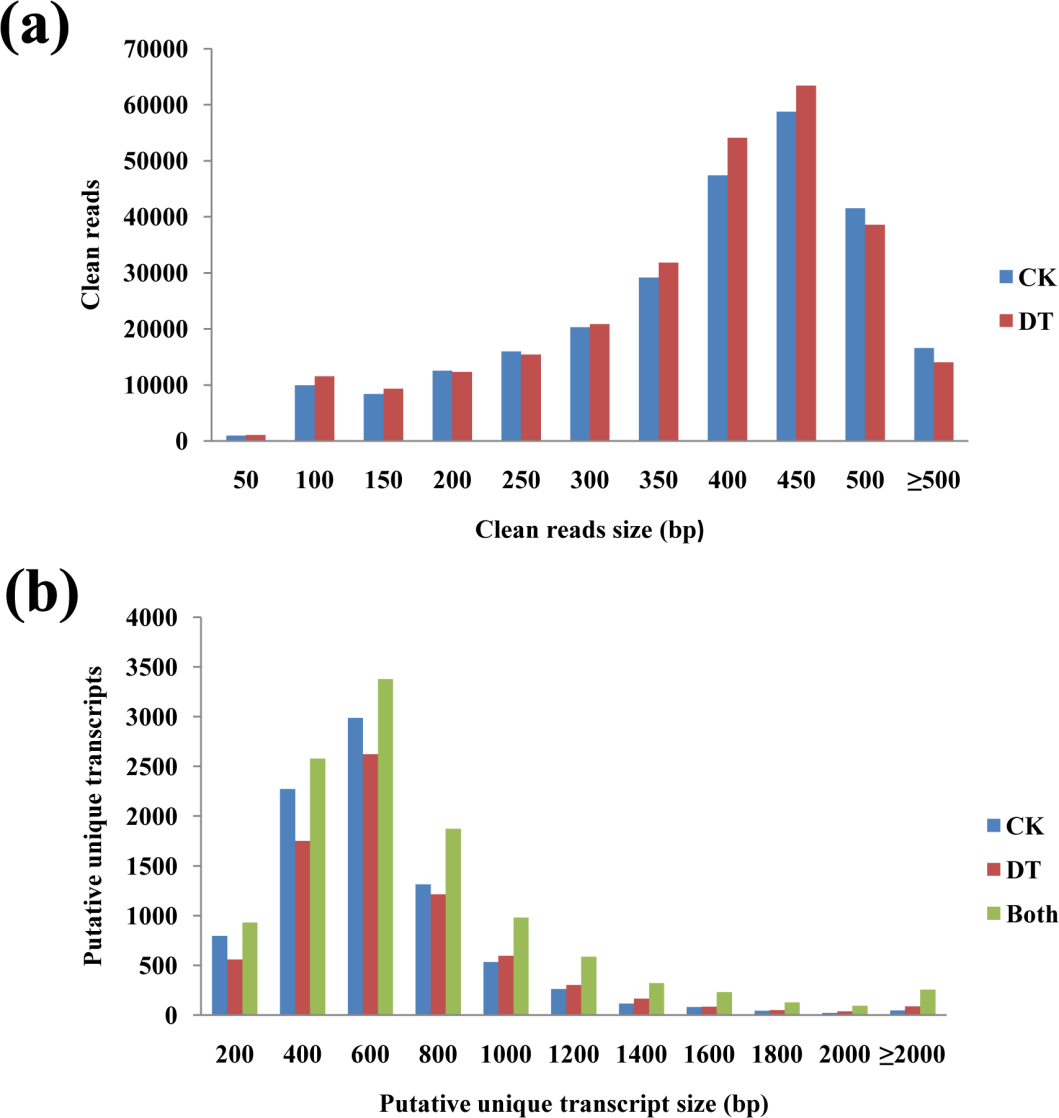
Size distribution of clean sequence reads and putative unique transcripts. Comparison of the length distributions of cleaned reads (a) and putative unique transcripts (b) from 454 pyrosequencing of control (CK) and drought-treated (DT) samples. Blue bars refer to CK data; Red bars refer to DT data; Green bars in graph B refer to PUTs from global (combined) assembly.

RNA sequencing was performed on total RNA isolated from leaves of the CK and DT groups, using the Roche 454 pyrosequencing platform (GS-FLX Ti). A total of 261,419 and 272,339 cleaned (filtered and trimmed) reads were generated from control and drought treated samples, respectively. Most of the sequence reads were distributed between 300 bp and 500 bp, peaking in length between 400 and 450 bp (Fig 1A). The minimum and maximum read lengths were 40 and 707 bp, respectively, with a median length of 387 bp and a mean length of 358 bp. The cleaned sequence data was deposited in the NCBI Sequence Read Archive (SRA, http://www.ncbi.nlm.nih.gov/sra) under accession number SRP026002. The cleaned reads from the two samples were assembled into PUTs separately, resulting in 8,480 contigs for CK and 7,474 contigs for DT, most of which ranged between 400 bp and 800 bp in length (Fig 1B). The average contig lengths were 526 bp (CK) and 579 bp (DT). When all of the sequences for the two groups were combined using TIGR Gene Indices clustering tools (TGICL) and the Contig Assembly Program (CAP3), 11,357 PUTs were obtained, with an average length of 618 bp (Table 1). The high quality assembled PUTs were deposited in the NCBI Transcriptome Shotgun Assembly Sequence Database (http://www.ncbi.nlm.nih.gov/genbank/tsa) under accession number SUB932387.

**Table 1:** Overview of the 454 pyrosequencing and transcript assembly results.

	CK	DT	Both
**Sequencing**			
**Total number of clean reads**	261,419	272,339	533,758
**Average length (bp)**	358.2	354.5	356.35
**Total bases**	93,643,151	96,548,448	190,191,599
**Assembly**			
**Percentage of aligned reads**	87.89%	90.58%	96.19%
**Percentage of aligned bases**	87.81%	90.19%	85.77%
**cDNA library-specific transcripts**	8,480	7,474	11,357
**Average length of PUTs** ^**a**^ **(bp)**	526	579	618

^a^ Putative unique transcripts (PUTs)

### Assessment of sequence read distribution

**Fig 2:**
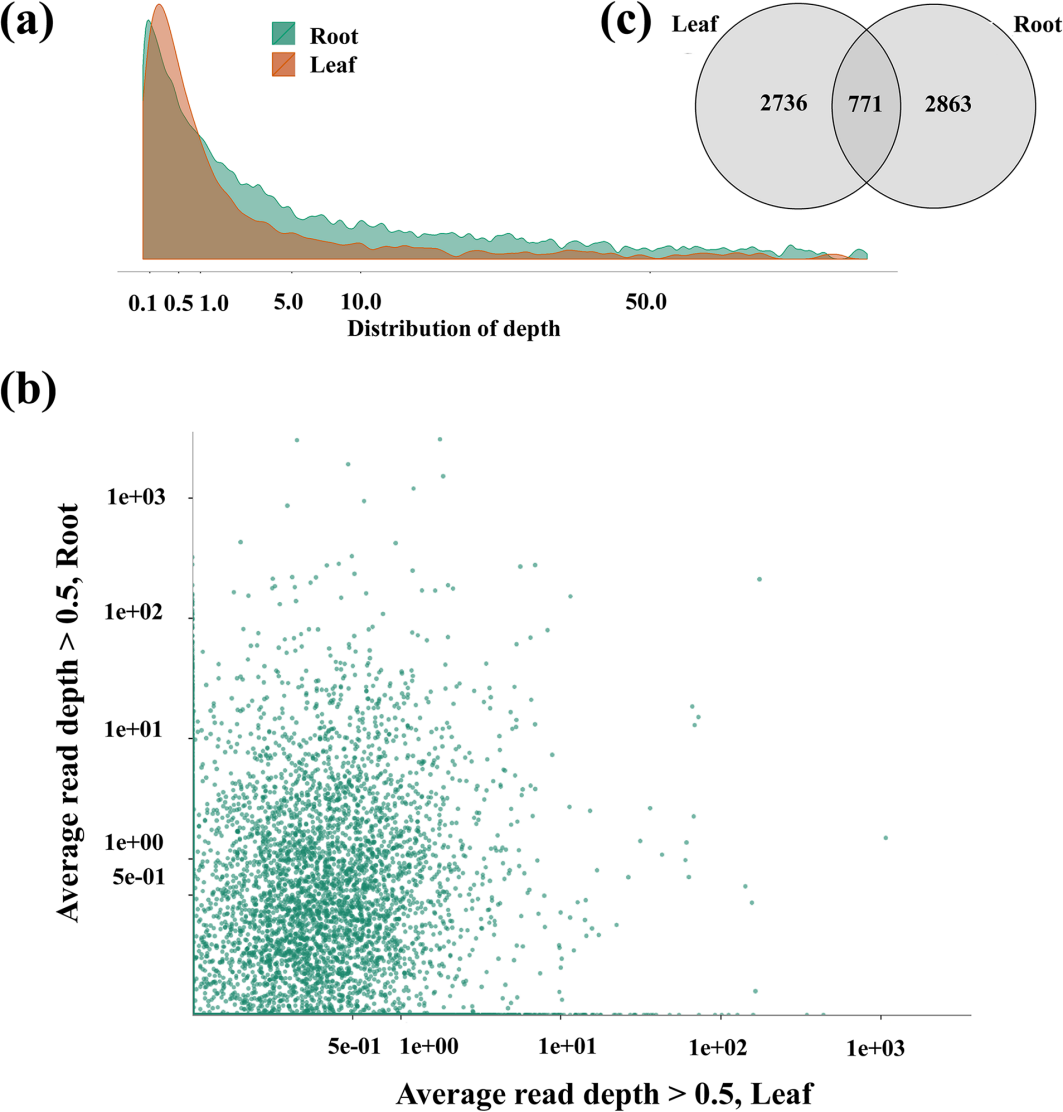
Distribution of sequencing depth among reads. (a) Similar univariate distributions of overall depth between root and leaf of sequence reads mapped to the *G*. *max* unigenes. (b) Random distribution of depths of reads for specific unigenes compared between leaf and root sequence data. (c) Venn diagram illustrating amount of overlap in *G*. *max* unigenes to which leaf and root reads mapped at average read depth > 0.5 in both tissues.

**Fig 3:**
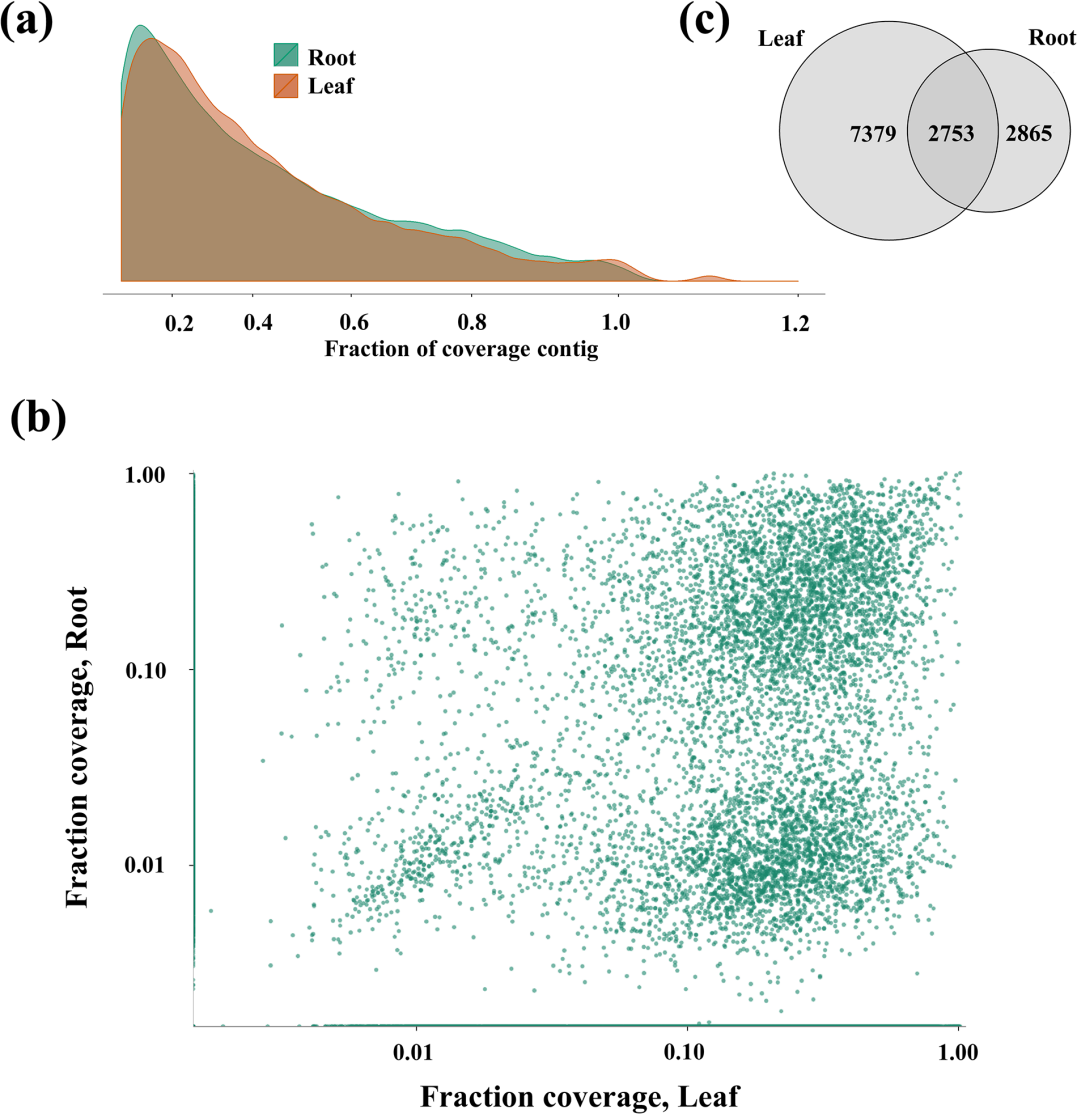
Distribution of gene coverage among reads. (a) Univariate distributions of unigene sequence coverage by root vs. leaf reads mapped to the *G*. *max* unigenes. (b) Distribution of unigene sequence coverage among leaf vs. root reads with >0.1 read coverage on average, showing categorical differences in coverage between the RNA sequence sources. (c) Venn diagram showing amount of overlap in unigene sequence coverage between root and leaf tissues.

In general, similar distributions of depth (number of reads per transcript contig) and coverage (number of genes identified amongst the reads) were observed when the previously published root RNA sequence reads (Zhou *et al*. [28]) and the leaf RNA sequence reads obtained in this study were mapped to the 35,982 *Glycine max* reference unigenes (Figs 2 and 3). However, some interesting contrasts emerged.

A generally univariate distribution of depth was observed overall for the mapping of root and leaf mRNA sequence reads to the *G*. *max* unigenes (Fig 2A). However, in spite of the highly similar overall distribution of depth of reads mapped to the *G*. *max* unigenes, there was little similarity in the number of root and leaf mRNA reads which mapped to specific *G*. *max* unigenes (each dot represents in Fig 2B represents a different unigene). We observed that of the 6,190 total *G*. *max* unigenes to which the average depth of reads mapped was greater than 0.5x, only approximately 12.5% (771) of the unigenes were represented in common among the reads from both tissues (Fig 2C).

The extent of gene coverage in the two EST data sets was also examined. The sequence reads from leaf tissue in this study mapped to 38.13% (13,720) of the *G*. *max* unigenes, while root tissue reads (from Zhou *et al*. [28]) mapped to 41.53% (14,943) of the *G*. *max* unigenes. In terms of the total number of *A*. *mongolicus* RNA sequence reads that mapped to the *G*. *max* unigenes, leaf-derived reads and root-derived were equally represented, at 48.4% and 49%, respectively. In contrast, 96.7% of the leaf-derived reads mapped to the *A*. *mongolicus* PUTs assembled in this study, while only 28.6% of root-derived reads mapped to the *A*. *mongolicus* PUTs.

Drastic differences were noted between the fraction of each reference unigene that the sequence reads from leaf and root tissues covered by mapping (Fig 3B). The fraction of unigene sequence coverage was simply calculated as the length of the combined consensus sequence of all reads mapped to a specific unigene, divided by the length of the respective unigene. Four distinct clusters of sequences emerged when the coverage by root vs. leaf sequence reads were compared for all cases in which at least 10% of the unigenes length was covered (Fig 3B). The unigenes clearly fell into all four possible clusters of root vs. leaf read coverage: unigenes for which the length of coverage was high by both root and leaf reads, unigenes for which the length of coverage was high by root reads but low by leaf reads, unigenes for which the length of coverage was low by root reads but high by leaf reads, and cases in which unigene length of coverage was low for both root and leaf reads. The numbers of unigenes in each of the clusters differed greatly however. The factor most affecting the difference in total counts of unigenes in each of the four classes was the disproportionate number of unigenes for which the length of coverage was high in leaf tissue mRNA reads, in contrast to the more uniform distribution of coverage of unigenes with reads from root tissue. The total numbers of unigenes covered at a minimum of 10% of their length by root vs. leaf reads is shown in Fig 3C. Of the 35,982 soybean reference unigenes, 12,817 (35.6%) had coverage length greater than 10% by either root and/or leaf reads. Of the 12,817 mapped unigenes, 2,753 (20.7%) were covered to 10% or greater length by mRNA from both root and from leaf tissues, while 3,865 (22.4%) were covered ≥10% length only by mRNA root reads, and a disproportionately large 7,379 (57.6%) of the unigenes were only mapped to ≥10% length with leaf reads.

### Functional annotation and classification of PUTs

**Fig 4:**
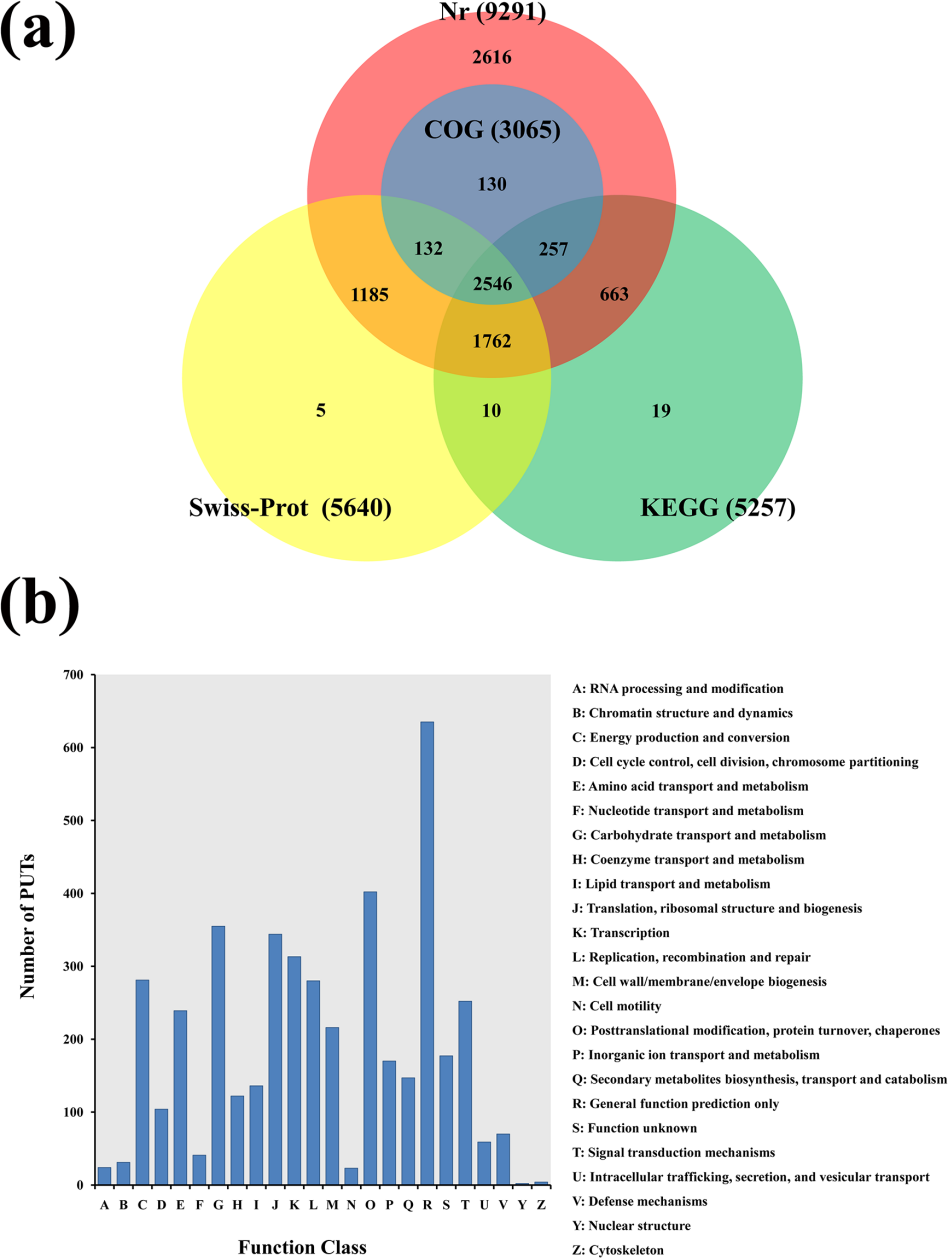
Annotation of *A*. *mongolicus* PUTs. (a) Venn diagram of the number of PUTs aligned by Blast to nr, Swiss-Prot, KEGG and COG databases. (b) COG functional classification of PUTs in *A*. *mongolicus*. A total of 11,357 PUTs were assigned to 24 classifications, as shown in the COG categories listed on the right side of histogram.

The *A*. *mongolicus* PUTs were annotated by alignment to gene and protein sequences in the public databases, as described in Methods. Homologous sequences for 9,375 (82.55%) of the PUTs were identified in at least one of the protein databases, and 2,546 (22.41%) of the PUTs could be aligned to all the databases. Protein sequence matches for the *A*. *mongolicus* PUTs were found in Nr (9,291, 81.81%), Swiss-Prot (5,640, 49.66%), KEGG (5,257, 46.29%), and COG (3,065, 26.99%). A total of 9,849 (86.72%) of the PUTs produced matches with entries in the Nt database, whereas homologs to the remaining 1,291 (11.37%) PUTs could not be identified. As might be expected, all 3,065 of the PUTs which aligned to the COG database also aligned to entries in the Nr database (Fig 4A). For the 1,291 PUTs that did not return any sequence matches, these may represent either non protein-coding sequences or genes encoding proteins with *A*. *mongolicus*-specific sequences (novel proteins). In addition, some of the *A*. *mongolicus* PUTs may have been too short to produce strong matches to sequences in the databases; given that 1,137 of the 1,291 un-matched PUTs (88.07%) were less than 500 bp in length.

We performed GO classification analysis of the *A*. *mongolicus* PUTs based on their Nr annotation information. A total of 7,070 (62.25%) PUTs were successfully assigned to one or more GO-terms (S1 Fig). The PUTs were classified as belonging to biological processes (4,457, 63.04%), cellular components (5,702, 80.65%), or molecular functions(5,643, 79.81%). For biological processes, the five largest categories were: ‘metabolic processes’ (4,070), ‘cellular processes’ (4,056), ‘response to stimulus’ (2,320), ‘biological regulation’ (1,659), and ‘pigmentation’ (1,554). For cellular components, the five largest categories were: ‘cell’ (5,061), ‘cell part’ (5,060), ‘organelle’ (4,166), ‘organelle part’ (1,607), and ‘macromolecular complex’ (1,037). For molecular function, the five largest categories were: ‘catalytic activity’ (3,669), ‘binding’ (3,407), ‘transporter activity’ (502), ‘structural molecule activity’ (206), and ‘transcription regular activity’ (165).

To identify possible functions of the PUTs during cellular metabolic processes, we performed a KEGG pathway analysis. The analysis resulted in 5,257 PUTs mapping onto 127 KEGG pathways. Among those mapped, 5,194 (98.80%) PUTs were related to metabolism, 1,182 (22.48%) PUTs corresponded to genetic information processing, 283 (5.38%) PUTs mapped to environmental information processing, 305 (5.80%) PUTs were classified as cellular processes and 231 (4.39%) PUTs belonged to organism systems.

To assess the integrity of our transcriptome libraries and evaluate the quality of the dataset, we performed COG classification of the *A*. *mongolicus* PUTs, which resulted in 3,065 matches (Fig 4A). COG classification placed the PUTs into 24 functional categories. The largest COG category was ‘General function prediction only’ (635), followed by ‘Posttranslational modification, protein turnover, chaperones’ (402), ‘Carbohydrate transport and metabolism’ (355), ‘Translation, ribosomal structure and biogenesis’ (344), and ‘Transcription’ (213). The least matches were in the categories of ‘Cell motility’ (23), ‘Cytoskeleton’ (4) and ‘Nuclear structure’ (2). Also, 177 (5.77%) PUTs were listed as ‘function unknown’ (Fig 4B).

**Fig 5:**
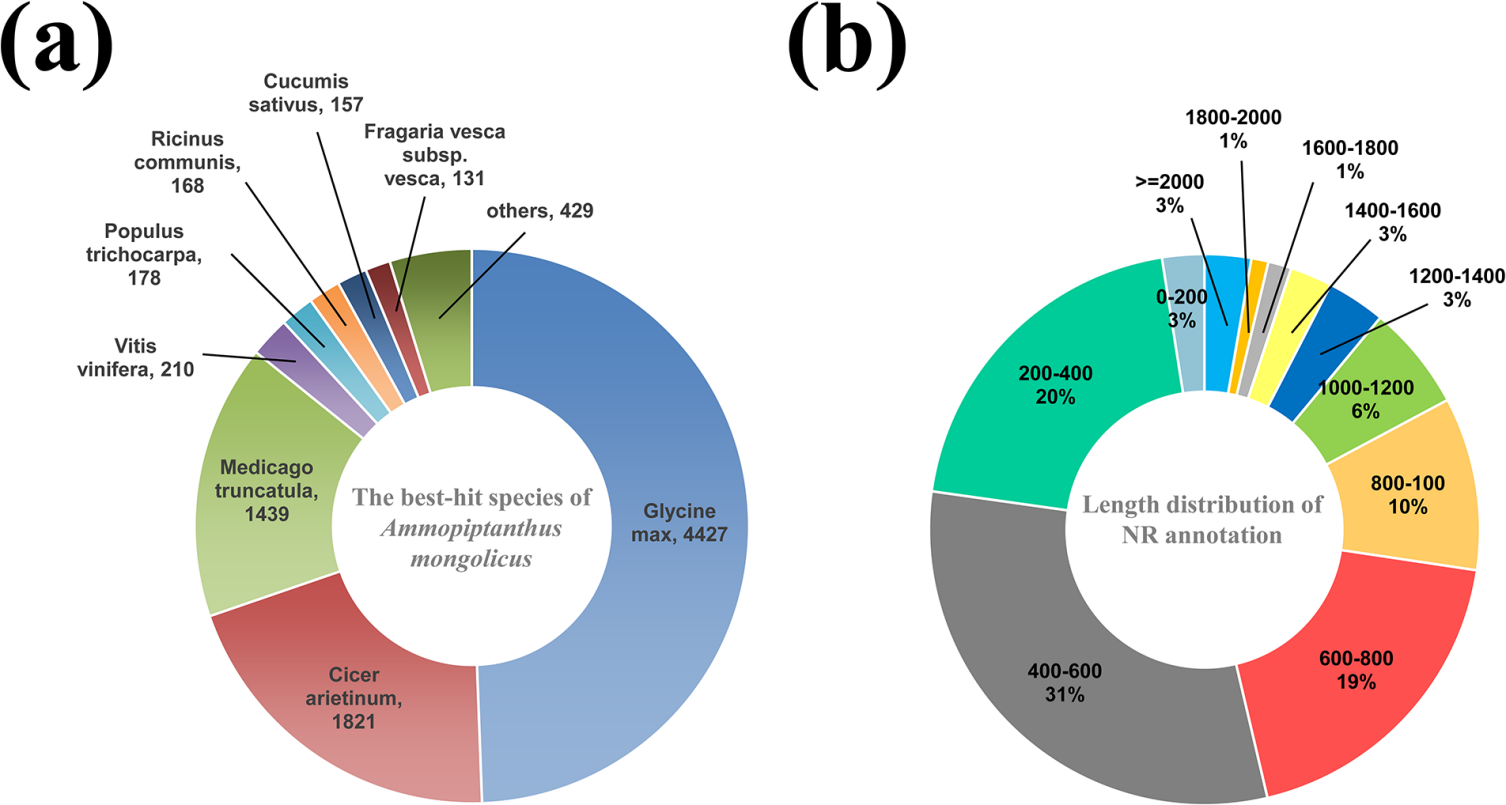
Species distribution of the BLAST alignment results for PUTs from *A*. *mongolicus* to the NR database. (a) Species distribution of the top BLAST alignment scores. (b) Distribution of PUTs sequence alignments lengths for matches to the NR database.

The Nr annotations showed that a total of 85.79% of the PUTs aligned with best BLAST scores to the three legume species with genome sequences—*G*. *max* (49.41%), *C*. *arietinum* (20.32%) and *Medicago truncatula* (16.06%) (Fig 5A). This conforms with the taxonomic classification of *A*. *mongolicus* in the *Leguminosae*. In addition, nearly one half of the PUTs had sequence alignment lengths to Nr database entries of over 600 bp (Fig 5B).

### Analysis of differentially expressed genes (DEGs)

**Fig 6:**
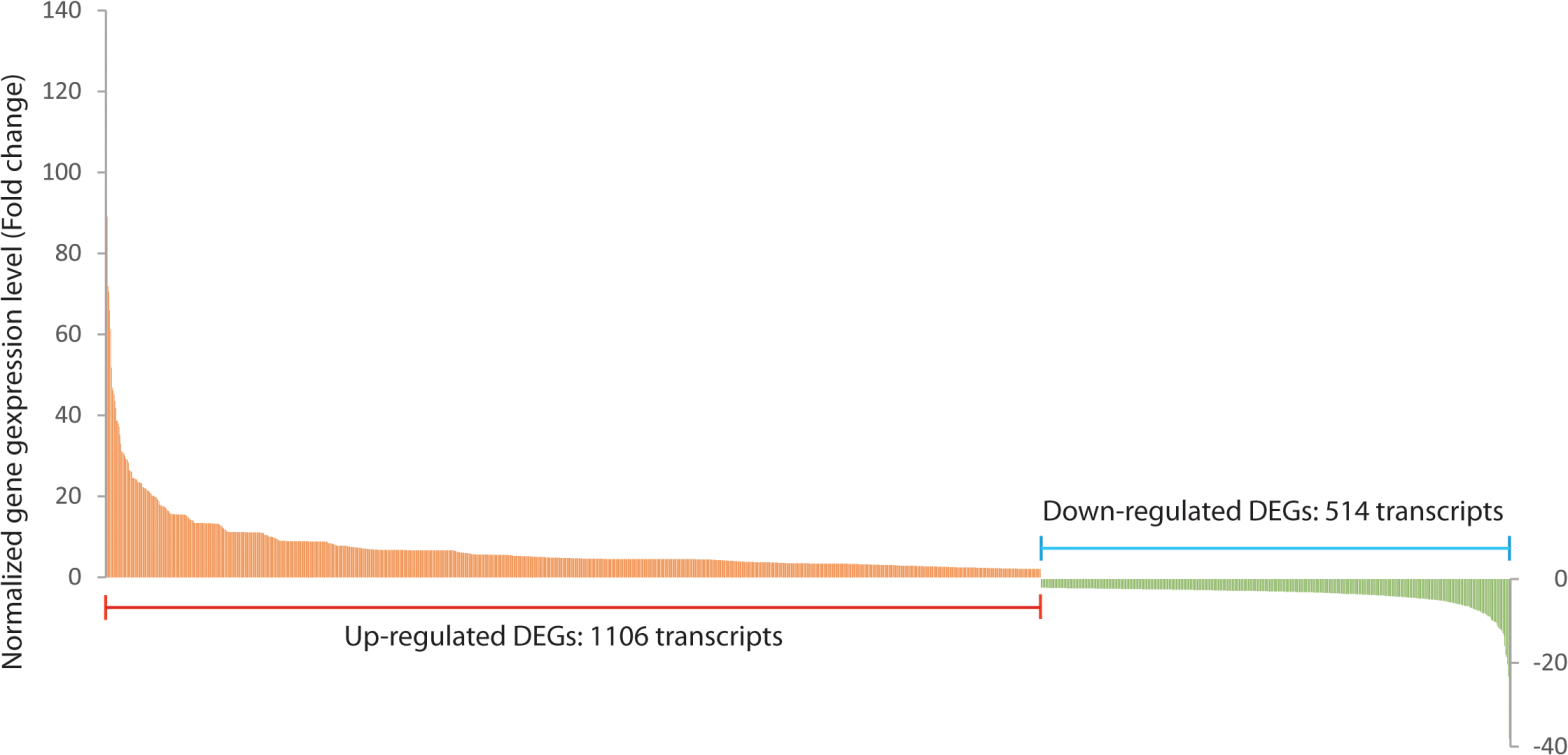
Differential expression analyses of the putative unique transcripts. DEGs were quantified by the RPKM method (cutoff value: FDR p-value < 0.01 and 2 fold change). The number of up- and down-regulated PUTs totalled 1106 and 514, respectively.

To identify differentially expressed genes in response to drought stress, and to compare expression levels, the RPKM method (Reads Per Kilobase per Million reads; [31]) was used. The Gene Expression software package in the CLCbio Genomics Workbench bioinformatics platform identified 1,620 PUTs as reliable DEGs (using a cutoff greater than 2 fold change and less than 0.01 of Kal’s z-test FDR p-value). Among the DEGs, 1,106 were significantly up-regulated in expression, while 514 showed down-regulation in the drought-stress treated samples (Fig 6). It is also noteworthy that 114 of the up-regulated genes and 202 down-regulated genes followed the same expression trends previously reported for cold-treated *A*. *mongolicus* plants [11]. The Nr annotations for these up-regulated genes included ‘CBL-interacting protein kinase 2-like’, ‘early light-induced protein’, ‘mitogen-activated protein kinase 19-like’, ‘WRKY transcription factor 23-like’, and ‘delta-1-pyrroline-5-carboxylate synthase-like isoform’. Annotations for the down-regulated genes included ‘protein early responsive to dehydration’, ‘mitogen-activated protein kinase 1’, ‘oxygen-evolving enhancer protein 1’, ‘iron-superoxide dismutase’, and ‘auxin-binding protein ABP19a-like precursor’ (S1 Table). A total of 1065 (65.74%) DEGs were successfully assigned to entries in the MIPS (Munich Information Center for Protein Sequence) Functional Catalogue database, of which 408 DEGs with strong functional annotations were classified into 19 main functional categories (S2 Fig).

**Fig 7:**
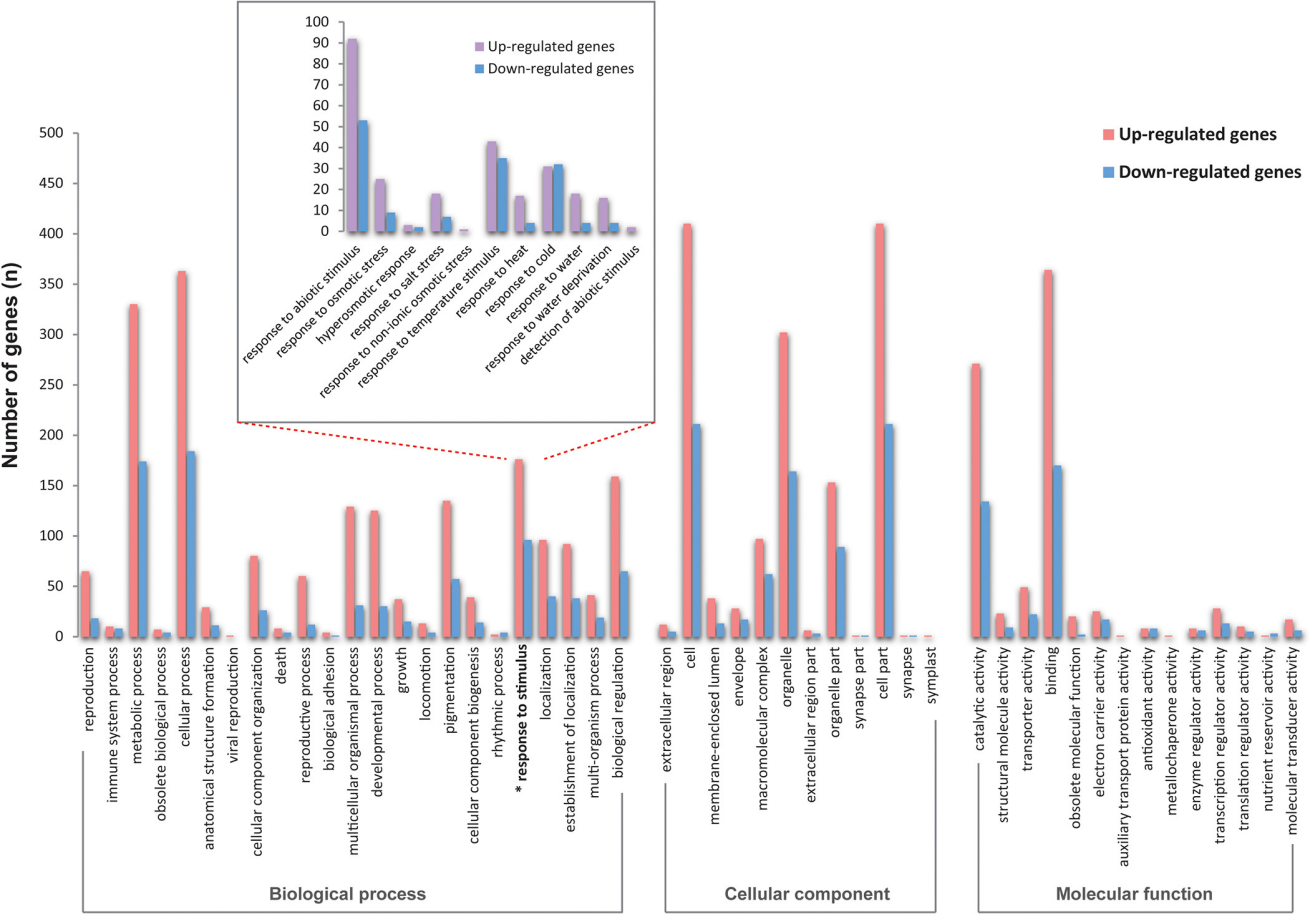
GO annotation of DEGs. From GO annotation results for all of the PUTs (S1 Fig), annotations of DEGs were summarized in the three main categories of biological process, cellular component and molecular function. The Histogram in the small insert box shows only the DEGs related to abiotic stress.

GO annotations were used to classify the DEGs as to the biological processes, cellular components, and molecular functions represented (Fig 7). For biological processes, the five largest categories of up-regulated and down-regulated DEGs (respectively in parentheses) were: ‘cellular processes’ (363:184), ‘metabolic processes’ (330:174), ‘response to stimulus’ (176:96), ‘biological regulation’ (159:65), and ‘pigmentation’ (135:57). For cellular components, the five largest categories were: ‘cell’ (410:211), ‘cell part’ (410:211), ‘organelle’ (302:164), ‘organelle part’ (153:89), and ‘macromolecular complex’ (97:62). And for molecular functions, the five largest categories of DEGs were: ‘binding’ (364:170), ‘catalytic activity’ (271:134), ‘transporter activity’ (49:22), ‘transcription regulator activity’ (28:13), and ‘electron carrier activity’ (25:17). Because this study was oriented towards understanding drought stress response in *A*. *mongolicus*, the genes identified as ‘response to stimulus’ in the third GO term levels were examined in more detail (Fig 7, small box). Among the 176 up-regulated genes and 96 down-regulated genes in the ‘response to stimulus’ category, most of the DEGs fell into the ‘response to abiotic stimulus’ category (92:53), followed by ‘response to temperature stimulus’ (43:35), ‘response to cold’ (31:32), ‘response to osmotic stress’ (25:9), ‘response to salt stress’ (18:7), ‘response to heat’ (17:4), and ‘response to water deprivation’ (16:4), respectively.

### Transcription factor analysis

**Fig 8:**
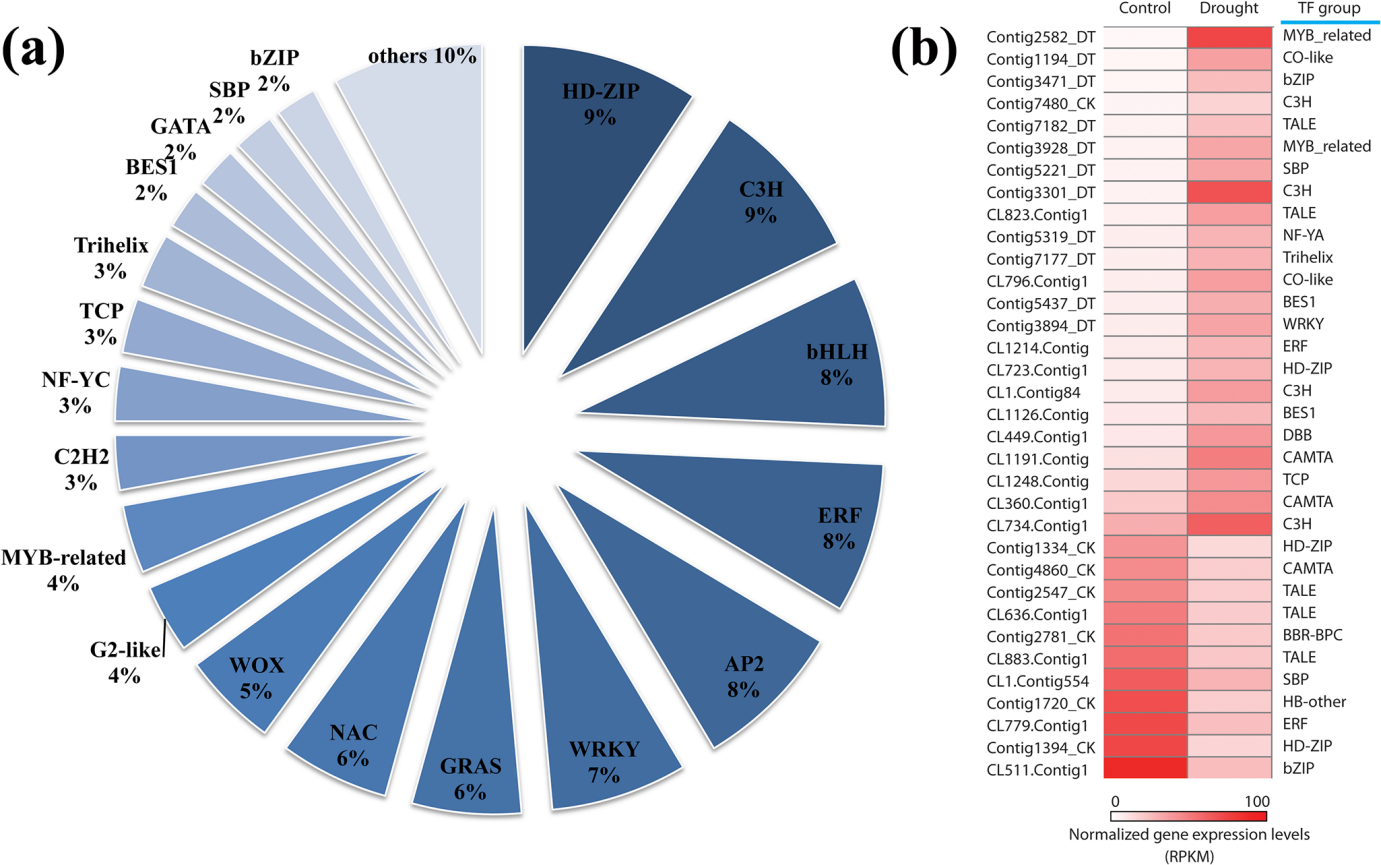
Distribution of transcription factors (TFs) among gene families. (a) Pie chart of percent of genes identified among TF gene families. (b) Heat map of significantly differentially expressed TFs under drought stress.

Transcription factors are widely involved in various biological processes, and play important roles in regulating genes expression in response to abiotic stresses. The domains of plant transcription factors were identified amongst the *A*. *mongolicus* PUTs, and classified according to gene families. A total of 144 PUTs were identified as encoding putative transcription factors in this study (Fig 8A). The largest transcription factor gene family observed was the HD-ZIP family (9.03%), followed by the C3H (8.33%), bHLH (7.64%), ERF (7.64%) and AP2 (7.64%) families. These five families accounted for a total of 40.28% of PUTs which were transcription factors. To demonstrate the dimensions of differential gene expression levels of transcription factors, a heat map of differential expression of transcription factors was constructed, for 23 transcription factors that were highly expressed under drought conditions, and 11 transcription factors that were down-regulated (Fig 8B). For TFs of interest to stress response, genes in the MYB, CO-like, C3H, NF-YA, Trilhelix, BES1, WRKY, DBB, and TCP families were specifically induced under drought conditions, whereas two TF groups, BBR-BPC and HB-other, were down-regulated.

### Quantitative real-time PCR

The transcriptome analysis results were validated by quantitative real-time PCR. A total of twenty candidate DEGs with specific annotations were selected, including genes encoding proteins of interest such as dehydrin, mitogen-activated protein kinase (MAPK), delta-1-pyrroline-5-carboxylate synthetase (P5CS), serine/threonine-protein kinase, temperature-induced lipocalin, RD22-likedehydration-responsive protein, salt-tolerance protein, transcription factor bZIP131, sodium/hydrogen exchanger, and auxin-binding protein ABP19a-like precursor. The qRT-PCR results were highly consistent with the expression profiles obtained by *in silico* digital expression analysis for these candidate genes (Table 2).

**Table 2:** Comparison of expression levels of drought responsive genes by *in silico* analysis and quantitative real-time PCR.

Putative unique transcript ID	Annotation (BLASTX)	*In silico* fold change (log2)	qRT-PCR fold change (2^-ΔΔCT^ ±SD)
CL231.Contig1	PREDICTED: beta-D-xylosidase 1-like	18.8	43.53±0.88
Contig2563_DT	early light-induced protein	11.135	11.06±2.41
CL2.Contig2	dehydrin [*A*. *mongolicus*]	8.501	11.24±0.81
CL1037.Contig1	PREDICTED: serine/threonine-protein kinase TOR-like isoform 1	7.755	10.54±1.05
Contig3471_DT	bZIP transcription factor bZIP131	7.399	2.36±0.87
CL73.Contig1	PREDICTED: plasma membrane ATPase 4-like isoform 1	6.656	9.99±1.16
Contig3094_DT	temperature-induced lipocalin	6.63	11.28±1.70
CL977.Contig1	PREDICTED: dehydration-responsive protein RD22-like	6.012	4.08±0.66
Contig1238_DT	Ribulose-bisphosphate carboxylase small chain, chloroplast precursor, putative	4.785	8.19±0.43
CL449.Contig1	salt-tolerance protein	4.472	2.82±0.23
CL47.Contig1	PREDICTED: delta-1-pyrroline-5-carboxylate synthase-like isoform 1	4.132	4.99 ± 0.79
CL372.Contig1	Outer membrane lipoprotein blc	4.052	2.75±0.45
CL1086.Contig1	mitogen-activated protein kinase 1	-2.189	-9.86±1.24
Contig75_CK	Cell wall-associated hydrolase	-2.246	-2.12±0.26
CL79.Contig1	photosystem II CP43 chlorophyll apoprotein	-2.88	-3.49±0.41
Contig3252_CK	PREDICTED: sodium/hydrogen exchanger 2-like	-2.897	2.81 ± 0.77
Contig831_CK	PREDICTED: dehydration-responsive protein RD22-like	-3.128	-7.31±0.88
Contig598_CK	PREDICTED: oxygen-evolving enhancer protein 1, chloroplastic-like	-3.534	-3.20±0.19
Contig1027_CK	serine hydroxymethyltransferase 5	-6.985	3.57 ± 0.19
CL1189.Contig1	auxin-binding protein ABP19a-like precursor	-8.781	-18.30±1.22

## Discussion

*A*. *mongolicus* is an ancient evergreen broadleaf shrub endemic to the semi-arid region of North-western China. *A*. *mongolicus* is able to survive in deserts where the average annual rainfall can be as low as 140 mm [32]. *A*. *mongolicus*’ drought adaptabilities include a strong root system to ensure absorption of any available water, and leaf structure with trichomes and sunken stomata that are believed to constrain transpiration. *A*. *mongolicus* should thus be an ideal species for the discovery of genes and physiological adaptations related to drought tolerance. Using the Roche-454 next generation sequencing technology, we successfully identified 11,357 unique transcripts in *A*. *mongolicus*, most of which were annotated by homologies to genes in other legumes, such as *G*. *max*, *C*. *arietinum*, and *M*. *truncatula*. Moreover, 1,620 (14.26%) transcripts showed significant expression differences between control and drought-stressed plants, with 1,106 being up-regulated and 514 being down-regulated. The DEG results were validated by qRT-PCR assay of the expression levels of 20 drought-responsive genes, selected randomly from the numerous DEGs. The DEGs were classified as being involved in drought-stress response and signal transduction.

### Sequencing strategy and data assessment

Zhou *et al*. [28] reported the analysis of the root transcriptome in *A*. *mongolicus* seedlings treated by irrigation with 20% PEG-6000 for 72 hours to induce water-stress. In contrast, we used a drought stress treatment as similar as possible to conditions experienced in nature, i.e. a long-term treatment (4 weeks) of plants in soil stressed to specific water potential values. Short vs. long term desiccation durations can produce different results in physiological and adaptive responses. Generally, plants respond to rapid dehydration by minimizing water loss or altering expression of certain genes to prevent dehydration damage. Under slowly developing water deficits, plants can survive against dehydration by optimizing their long term acclimation responses, including changes to shoot physiology and resource allocation [33]. As soils become dry, signals from the roots are transported via the xylem to leaves, resulting in reduced water loss and decreased leaf growth, followed by changes in abscisic acid (ABA), pH, cytokinins, ethylene precursors and other factors related to stress signaling processes[34–35]. Thus, while it is appropriate to study responses in root tissues under short-term water-stress conditions, above ground tissues are valuable in research on longer-term responses to drought stress. The treatment length was chosen from results of preliminary experiments in which physiological status and expression of candidate drought-response genes were assessed by qRT-PCR at 2, 3 and 4 weeks intervals. The 4 week period produced most consistent results, without affecting plant growth or phenotype (S3 Fig).

Zhou *et al*. [28] assembled 29,056 unique transcripts (including both 15,713 transcript contigs and 13,883 unique unassembled singlet reads) from root RNA sequences after short term osmotic-stress, from which they selected 27 candidate genes from specific GO categories such as ‘response to osmotic stress’, ‘response to oxidative stress’, ‘response to hormone stimulus’, and ‘response to light stimulus’. We considered it important to compare expression of a broader set of functional categories in the shoot and root transcriptomes. Thus we selected candidate genes from several general response to stimuli categories including ‘response to osmotic stress’, ‘response to salt stress’, ‘response to temperature stimulus’, ‘response to heat’, ‘response to cold’, ‘response to water deprivation’ and ‘detection of abiotic stimulus’. In addition, we studied transcription factors to identify potential regulators of drought response. Only two of the 27 candidate genes identified by Zhou *et al*. (sdq_isotig00259 and sdq_isotig02931) overlapped with the 1,106 up-regulated and 514 down-regulated DEGs identified in this study.

Our PUT set of 11,357 contigs is similar in number to the 15,713 contigs assembled by Zhou *et al*. [28], given that singlets were not included in our PUT totals. We did not include singlets to avoid over-representation of genes represented as multiple small fragments among the contigs, which can bias differential gene expression results. Also individual reads can be more difficult to assign to specific members of gene families than contigs, complicating transcriptome analysis.

More PUTs may have been expected from the transcriptome assembly, however, given the large number of high quality sequence reads obtained (over 190M) and the fact that many genes are normally expressed in leaves. Strong abiotic stresses, such as drought, may transiently suppress transcription, however, as well as result in degradation of the existing transcriptome. This could result in a smaller, less complex transcriptome, if *de novo* transcription of stress-response genes are then predominant, at least transiently. Perhaps differences in complexity of transcriptomes among abiotic stress studies may relate in part to stress levels, for example if shorter, less intense stress treatments have less effect on suppression of gene expression. Furthermore, at the time that the stress treatment commenced, the leaves that we sampled were already fully developed, at which time only house keeping genes may have been active. These factors are difficult to assess with our *A*. *mongolicus* data however, without a genome or complete gene set for *A*. *mongolicus* to serve as a reference.

The sequencing was conducted in this study with RNA isolated from leaves pooled from among the replicate plants within each treatment. Pooling of replicates was chosen to maximize the depth of sequencing possible within each treatment, and to capture general drought responses in *A*. *mongolicus*. However, the approach did not capture variation that may have existed among the *A*. *mongolicus* seedling genotypes, nor the response of other above ground tissues in the seedlings. Our analyses from this study can only infer general differences in gene expression levels in leaves of control vs. treated plants. Thus further investigation into genetic- and tissue-based differences in gene expression under stress conditions is warranted for future studies.

### Comparative genomics analysis

The MIPS functional catalogue assigned 25 DEGs as being involved in ‘transcription’, of which 18 were strongly up-regulated. Also, 26 DEGs were identified to be related to ‘signal transduction’, of which 18 were up-regulated and 8 down-regulated. Several of these DEGs have homologues in *Arabidopsis*, such as *AtCIPK9* (CL736.contig1), *AtCIPK11* (Contig7006_DT) and *AtCIPK12* (CL1014.contig1), which are known to be stress related regulators of downstream target genes [36, 37]. We also noticed that 584 DEGs were annotated as ‘Classification not yet clear-cut’ or ‘Unclassified protein’ when aligning to *Arabidopsis* database. However most of these genes have homologues in other species, including *G*. *max*, *M*. *truncatula* and *C*. *arietinum*. It is noteworthy that three significantly up regulated DEGs were annotated as genes associated with asparagine synthesis-*AtASN1* (CL828.Contig1 and CL759.Contig1) and *AtASN3* (Contig6402_DT). As is known, osmotic stresses lead to Asn accumulation and/or Asn-synthetase induction [38]. ASN is a small gene family encoding the synthetase of asparagine, which was reported to be induced during periods of carbon starvation in maize and rice [39]. Also, *TaASN1* from wheat was reported to be up-regulated by osmotic stress and ABA treatment [40]. The enhancement of asparagine synthesis and accumulation suggest the nitrogen metabolism and transport may be induced to ameliorate the deleterious effects of longer-term drought stress in *A*. *mongolicus*. On the other hand, some genes not previously reported only in association with ABA- or salt-tolerance [41,42] but not in drought stress were identified in *A*. *mongolicus*, such as FAD6 (Fatty acid desaturase-6) and CPN20 (chaperonin 20). Further studies with these genes ae required to determine if they represent drought-specific mechanisms however.

### *A*. *mongolicus* response to drought stress

Plants respond to drought stress in various physiological and morphological ways, which includes the accumulation of proline, expression of Aquaporins (AQPs), and biochemical alterations in the cell wall [43]. Among the DEGs identified in this study, 145 were classified in the GO category of ‘response to abiotic stimulus’ (Fig 7), including genes which encode enzymes for the synthesis of osmoregulatory substances, such as proline which is known to increase in response to osmotic stress [44]. It has been previously reported that delta 1-pyroline-5-carboxylate synthetase (P5CS) for proline synthesis is upregulated by drought stress [45]. Similarly, homologs of genes encoding P5CS (CL47.Contig1, CL449.Contig1) were observed to be highly expressed in *A*. *mongolicus* under drought stress in this study. In addition, genes encoding membrane transport proteins such as AQPs and plasma membrane ATPase (CL73.Contig1) were also significantly induced in drought-treated samples. Five DEGs related to AQPs were identified, among which three (Contig762_DT, Contig3396_DT, CL677.Contig1) showed significant up-regulation and two (CL679.Contig1, Contig3627_CK) were down-regulated between control and drought conditions. Aquaporins belong to a large protein family, with members regulated differentially during drought stress, which is consistent with important roles in water homeostasis [46]. Furthermore, numerous DEGs with high expression levels were observed which may be related to specific structural adaptations that enable *A*. *mongolicus* to survive severe drought in deserts, such as cell wall-associated hydrolases (CL1.Contig77, CL1.Contig411, CL1.Contig510, *et al*.). Cell wall-associated hydrolase genes were reported to function in altering cell wall extensibility and other cell wall modifications, such as the accumulation of compatible extra-cellular solutes [47, 48].

### Photosynthesis

As a complex metabolic process, photosynthesis is well studied and known to be sensitive to drought stress [49]. The result of our metabolic pathway analysis showed 29 DEGs involved in photosynthesis, including 13 subunits (PsaB, PsaD, PsaE, PsaF, PsaG, PsaH, PsaJ, PsaK, PsaL, PsaN, PsaO) in the photosystem I (PSI) complex. Most of the differentially expressed Psa subunit genes were down-regulated in response to drought stress, with the exception of PsaB, PsaD and PsaJ. There was also one DEG (Contig1238_DT) related to ‘Ribulose bisphosphate carboxylase small chain’, which was found to be up-regulated under drought conditions. Even though Rubisco expression has been previously observed to be down-regulated under osmotic stress in many plant species [50, 51], studies with sugarcane and cucumber [52, 53] reports up-regulation of Rubisco, similar to our finding with *A*. *mongolicus*. Furthermore, change in expression of Rubisco protein has been identified in the halophyte *Suaeda aegyptiaca* under salt stress [54]. Overall, these results suggest that drought stress response in *A*. *mongolicus* may have similar mechanisms as salt stress response, in keeping with previous observations on similarities among responses to abiotic stresses in plants (1, 12, 35, 53, 63).

In addition, 10 DEGs in *A*. *mongolicus* identified in this study were associated with the KEGG term of ‘Photosynthesis—antenna proteins’, including genes encoding the Light-harvesting complexes (LHCs). It is noteworthy that all ten *A*. *mongolicus* DEGs related to LHCs were down-regulated under drought stress in this study. It has been reported that some members of the CAB superfamily (Lhca and Lhcb genes) are down-regulated by high light stress, and that overall about 70% of genes observed to be down-regulated in high-light were also down-regulated by drought [55]. Studies in *A*. *thaliana* indicated that photosynthetic response to drought stress is highly complex, involving alterations of a multitude of genes, resulting in the improvement of stress tolerance [33]. Therefore, *A*. *mongolicus* may have specific photosynthesis responses to drought related to being a xerophyte shrub with thick evergreen leaves.

### Protein phosphorylation and dephosphorylation

The complex process of signal transduction is an important component in the response to drought stress. Protein phosphorylation and dephosphorylation play an important role in signal transduction cascades, involving protein kinases and phosphatases [56]. In this study, a total of four DEGs (two up-regulated and two down-regulated) were found to be involved in mitogen activated protein kinases (MAPK) cascades. Evidence from animal and yeast studies suggests that the MAPK superfamily performs critical roles in adaptive responses to osmotic stress [57]. Kiegerl *et al*. reported the isolation and characterization of alfalfa MAPK kinase, and that it was activated by salt stress [58]. Recently, a series of tobacco MAPK genes have been identified including six which were shown by qRT-PCR analysis to be induced by drought treatment [59]. One DEG (CL1037.Contig1, 7.755 fold) observed in this study encoded a serine/threonine-protein kinase TOR-like isoform 1.TOR kinase expression can be regulated by both sugar abundance and starvation, connected to many energy-consuming cellular outputs [60]. Therefore, TOR is believed to play important roles in plant developmental and metabolic processes [61]. Sucrose non-fermenting1-related protein kinase 2 (SnRK2) is known to be related to plant sugar signaling via phosphorylation [62]. Wheat SnRK2 members, like *TaSnRK2*.*4*, *TaSnRK2*.*7* and *TaSnRK2*.*8* were identified as being involved in abiotic stress responses [63]. *Arabidopsis* transformed with *TaSnRK2*.*4* was reported to have significant improvement in drought, high salinity and cold stress resistances, as supported by physiological experiment data [64]. In this study, two DEGs (Contig2630_DT and Contig5982_DT) associated with SnRK2s were found to be significantly up regulated in *A*. *mongolicus*.

### ABA signaling pathway

Considering the crucial role that ABA plays in abiotic stress responses, another focus of this study was transcription factors and genes related to the ABA signaling pathway. It has been reported that dehydration activates both ABA-dependent and ABA-independent stress signaling pathways [65]. In the ABA-dependent signaling pathway, the bZIP domain in basic leucine zipper family transcription factors has been shown to interact with a conserved ABA-responsive, cis-acting element known as ABRE (ABA-responsive element) in promoter regions [66]. In this study, two DEGs in *A*. *mongolicus* (Contig3471_DT and CL511.Contig1) homologous to *G*. *max* bZIP protein encoding genes were identified. Interestingly, the Contig3471_DT gene was observed to be up-regulated, while the CL511.Contig1 gene was down-regulated in response to drought. It is also known that transcription factors such as MYB and MYC may be activated, and interact with certain cis-elements, thus inducing the expression of drought responsive genes [67]. We observed two DEGs related to MYB transcription factors that were induced by drought stress. However, no MYC-related DEGs were identified in *A*. *mongolicus* in this study. In the alternate, ABA-independent signaling pathway, gene expression under drought stress is not related to ABA levels [68]. The dehydration-responsive element (DRE) is a 9-bp conserved sequence, TACCGACAT, that has been observed in the promoter region of drought-responsive genes [69]. Commonly, the transcription factors of DRE-binding proteins contain the AP2/EREBP domain, such as DREB1/CBF and DREB2 (DRE binding protein) [70, 71]. In this study, a number of *A*. *mongolicus* PUTs were observed that match the AP2/DREBP domain, 11 of which showed significant differential expression under drought. The soybean *GmDREB2* protein has been reported to promote the expression of downstream genes to enhance drought and high salinity tolerance in transgenic *Arabidopsis* [72]. The WRKY gene (Contig3894_DT) identified in our study, which was highly expressed under drought stress, had not been previously reported in *A*. *mongolicus*. Thus further functional characterization of this gene is warranted, similar to studies of overexpression of soybean GmWRKY genes in *Arabidopsis* in which plants were reported to be more tolerant to cold (*GmWRKY21*), salt (*GmWRKY54* and *GmWRKY21*) or drought (*GmWRKY54*) stress than wild-type plants [73, 74]. WRKY TFs were suggested to play a key role in ABA and drought-responsive signalling networks [75]. Even though *WRKY23* was originally identified as a plant-parasitic nematode-inducible gene, current studies also indicated that *PtWRKY23* is involved in redox homeostasis and cell wall-related metabolism [76]. The function of the *WRKY23* gene in *A*. *mongolicus* under drought treatment remains to be determined through further research, including transgenic studies in *Arabidopsis* or a leguminous model such as *M*. *truncatula*.

## Conclusion

In this report, we provided a comprehensive transcriptome analysis of *A*. *mongolicus* under drought stress, incorporating data and findings from previous reports as well as new experimental findings. All of the putatively unique transcripts were annotated, and drought-responsive DEGs were classified as to functional categories and metabolic pathways. Many candidate genes potentially involved in drought stress in *A*. *mongolicus* were identified.

The one month water deprivation regime applied in this study generated a relatively small decrease in the leaf water potential in the drought-tolerant *A*. *mongolicus* plants indicating that the plants did not experience the degree of physiological stress that might be expected in drought-sensitive plants under the same conditions. To create greater physiological stress levels, a longer and more severe water-deprivation regime would have been required, which may have elicited a stronger gene expression response in the *A*. *mongolicus* plants as well. However, in this study we were interested in learning how *A*. *mongolicus* plants tolerate water deprivation conditions that would damage most other species, and if there is a gene expression component associated with that, rather than determining what levels of water stress would produce the same physiological and gene expression responses in *A*. *mongolicus* as in drought-sensitive species exhibit under lower stress levels. All species, even desert plants, will be damaged or perish at some point after prolonged drought conditions. However response to extreme, damaging stress conditions may not reflect inherent tolerance mechanisms in desert plants. The fact that *A*. *mongolicus* plants in this study did demonstrate many of the typical changes in gene expression in response to water stress conditions, relative to controls, documented that the treated *A*. *mongolicus* plants did perceive the stress conditions, even though the plants were not yet showing many signs of physiological stress. Perhaps, in part, *A*. *mongolicus* adaptation to xeric conditions involves a more rapid gene expression response, or the ability to respond to lower levels of physiological stress, than in more drought-sensitive species. It would be interesting in future research to investigate if the upstream transcription factor DEGs which we uncovered in this study play a role in the priming of expression of defence-response gene expression pathway genes in *A*. *mongolicus*. Drought-tolerance mechanisms in *A*. *mongolicus* may also include structural and constitutive adaptations of equal or greater importance than temporal gene expression changes, which would also be worthy of additional study.

All data in this study have been publically archived for future research. We hope that this report will lead to more research on the functional identification of drought-responsive genes and mechanisms in *A*. *mongolicus*, to enrich our knowledge of the basis of stress tolerance in desert-adapted plants and facilitate the improvement of drought resistance in other plants.

## Materials and Methods

### Plant material

This study was approved by the National Engineering Laboratory for Tree Breeding. Seeds of *A*. *mongolicus* were identified and collected by Dr. Chao Liu (Beijing Forestry University) from the Sand Control Station of Wuhai (39.62°N, 106.76°E), in Inner Mongolia Autonomous Region, China. After sterilization using 70% ethanol for 30s and 10% sodium hypochlorite for 20min, the *A*. *mongolicus* seeds were planted in MS (Murashige-Skoog) medium. After two weeks of growth, the seedlings were transplanted to plastic pots (10 cm ×10 cm × 10 cm) containing peat/sand/vermiculite (7:2:1), in the nursery of Beijing Forestry University (BJFU) (40.0°N, 116.6°E). The transplanted seedlings were separated into two groups: control group (CK) and drought-treated group (DT). Controls were watered to field capacity every day. For the DT group, the water stress treatment was implemented by stopping watering on the first day and withholding water for the next two to four weeks, which simulated a natural drought process (S3 Fig). We performed the qRT-PCR with four drought related genes identified by our previous study [27] to show the best regimen for drought treatment. The leaf water potential (LWP) of the *A*. *mongolicus* seedlings was measured with the PSYPRO water potential system (WESCOR, INC, USA). Values of LWP were measured as MPa. Three biological replications were performed. Photographs were taken to document general plant condition, which did not decline noticeably, at 2, 3, or 4 weeks treatment (S3 Fig). Finally, leaves of both groups were collected at the same time point for RNA extraction.

### RNA isolation

Leaves collected from each group of seedlings were pooled to form the control (CK) sample and four weeks drought (DT) sample for RNA isolation. Total RNA was isolated using a CTAB procedure [77]. Tissues of CK and DT samples were separately ground in liquid nitrogen and the powder dispersed in CTAB buffer. After two extractions using chloroform-isoamyl alcohol mix (v/v 24:1), the total RNA was precipitated with LiCl_2_. To ensure the quality and concentration, the precipitate was suspended in SSTE buffer (0.5%SDS, 10mM Tris-HCl, 1mM EDTA and 1M NaCl), and extracted twice again with chloroform [20]. Finally, the RNA was precipitated with 3 M sodium acetate and dissolved in 100 μl diethypyrocarbonate (DEPC)-treated water. For each sample, we conducted three extractions of total RNA. The total RNA yield and purity were measured using a NanoDrop 2000 (Thermo Fisher Scientific). The A260/A280 ratios for both samples ranged from 1.9 to 2.1. The quality and concentration of all RNA samples was examined with an Agilent 2100 Bioanalyzer (Agilent Technologies) which showed no sign of degradation. Finally, mRNA was purified from the total RNA using the Oligotex-dT30<Super> mRNA Purification Kit (Takara, China).

### cDNA preparation and 454 pyrosequencing

First-strand cDNA synthesis was performed with 5 ug of mRNA for each sample, using the Just cDNA Double-Stranded cDNA Synthesis Kit (Agilent Technologies, USA, catalog #200453). Double stranded cDNA was synthesized following the manufacturer’s recommendations. The cDNA was then dissolved in sterile distilled water, and the concentration and quality was determined using an Agilent 2100 Bioanalyzer. The cDNA libraries for sequencing were constructed according to the manufacturer’s manual (Roche, 454), and one half plate of FLX sequencing was performed for each of the cDNA libraries. Both the cDNA library construction and 454 pyrosequencing were conducted in the Genomics Center at the Pennsylvania State University.

### Sequence read processing and *de novo* assembly

The 454 raw sequencing reads were processed before assembly. Roche library adaptors, ribosomal RNA, low quality scored bases, and contaminants that may negatively affect clustering and assembly were removed. The high quality, filtered reads from each sample were then assembled using SeqManNgen sequence assembler v2.1 (DNASTAR, Inc.) software in cDNA mode with default parameters. *De novo* assembly was necessary due to lack of genome information on *A*. *mongolicus*. Finally, the contigs from the two libraries were combined to form a single set of non-redundant transcripts. The contigs were clustered based on pairwise sequence similarity, using TGICL and CAP3 programs [78]. This provided a set of non-redundant consensus sequences subsequently referred to Putative Unique Transcripts (PUTs), used for annotations and gene expression analyses. In addition, previously published RNA sequence data from *A*. *mongolicus* drought-treated and control root tissues was downloaded from the SRA database at NCBI (Zhou *et al*., accession number SRX142053) [28]) totalling over 671,000 reads. The reads were filtered to remove low quality score bases and adapter remnants.

### Sequence read mapping

The cleaned sequence reads were mapped to a set of 35,982 *G*. *max* unigenes retrieved from the NCBI UniGene repository (ftp://ftp.ncbi.nih.gov/repository/UniGene/). Both the drought-treated and control root tissue sequence reads downloaded from NCBI (Zhou *et al*. [28]) and the drought-treated and control leaf tissue sequence reads from this study were mapped against the *G*. *max* unigenes. Read mapping was performed using CLCbio Genomics Workbench 7.5 with the affine gap scores and unrestrictive local alignment parameters set at the same value for the mapping of the root and leaf reads. Similarly, reads from root and leaf data sets were mapped against the *A*. *mongolicus* PUTs generated in this study. The resulting distributions of read depth and unigene coverage from mapping were plotted as R.Venn diagrams using an R script with the following settings: leafDepth<- subset(GmaxMerge, Average.coverage.y> 0.5); rootDepth<- subset(GmaxMerge, Average.coverage.x> 0.5); depth.venn<- venn.diagram(list(Leaf = leafDepth$shortName, Root = rootDepth$shortName), fontface = "bold", fill = "grey", alpha = 0.5, scaled = T, "DepthVenn.tiff"); leafCover<- subset(GmaxMerge, coverage.y> 0.1); rootCover<- subset(GmaxMerge, coverage.x> 0.1); cover.venn<- venn.diagram(list(Leaf = leafCover$shortName, Root = rootCover$shortName), fontface = "bold", fill = "grey", alpha = 0.5, scaled = T, "CoverageVenn.tiff"). For Figs 2C and 3C, read depth was calculated as the value of total reads divided mapped by the number of reads in the sample. Read coverage was calculated as the total basepairs of a unigene contig covered by at least one read divided by the length of that contig.

### Functional annotation and classification

The 11,357 PUTs from *A*. *mongolicus* leaf sequences were annotated by alignment to the following protein databases: [Nr (NCBI non-redundant protein database), Swiss-Prot, KEGG (Kyoto Encyclopaedia of Genes and Genomes database), and COG (Clusters of Orthologous Groups of proteins)] using the BLASTX method (E-value < 1E-5). The PUTs were also annotated by alignment to the nucleotide sequence database Nt (NCBI non-redundant nucleotide sequence database) by BLASTN (E-value < 1E-5). The annotations with the highest sequence similarity (BLAST scores) were chosen for functional categorization of the PUTs. To gain a better understanding of the distribution of gene functions at the macro-level represented in the set of PUTs, we performed GO annotation using the Blast2GO program [79], and GO classifications using the WEGO software [80], using the best Nr-based annotations. From the KEGG-based alignments, pathway annotations were constructed, to provide a better understanding of the metabolic pathways involved in drought stress-response in *A*. *mongolicus* [81]. Also, the protein-coding regions of the PUTs were predicted and translated into amino acid sequences, based on the best BLAST alignments. For those PUTs that could not be aligned to any of the above databases, we used ESTScan [82] to predict possible coding regions and direction of translation.

### Analysis of differentially expressed genes (DEGs)

Differentially expressed drought-responsive genes were identified and quantified using CLCbio Genomics Workbench software. The RPKM (reads per kb per million reads) method was used to calculate the fold-change of the expression levels of each PUT between control and treated samples [31]. For statistical analysis, Kal’s Z-test was used to conduct pair-wise comparisons of gene expression proportions of specific tags in the sequence reads between individual samples. PUTs with the absolute log-ratio value of ≥ 2 and with p-value cutoff of less than 0.01 of FDR p-value correction were selected as reliable DEGs. DEGs were assigned to *Arabidopsis* gene models (TAIR ver. 10, http://www.arabidopsis.org/), using BLASTx (cutoff E-10). The gene ids were then entered into the “FunCat” Functional Catalogue Database at the Munich Information Center for Protein Sequence (MIPS) classification system (http://www.helmholtz-muenchen.de/en/ibis/resourcesservices/services/funcat-the-functional-catalogue/index.html).

### Metabolic pathways and transcription factors analysis

DEGs were assigned to the primary cellular biochemical pathways and signal transduction pathways by comparison of the BLAST results to the KEGG database. The predicted protein sequences were also aligned to the Plant Transcription Factor Database (http://plntfdb.bio.uni-potsdam.de/v3.0/) by BLASTx, and the PUTs classified according to TF gene families. Candidate genes associated with drought stress were selected from among the KEGG and TF family assignments for validation.

### Quantitative real-time PCR analysis

The expression levels of the selected candidate DEGs were confirmed by qRT-PCR analysis. The cDNA for the CK and DT samples was produced from approximately 1 μg of total RNA from each group using M-MLV Reverse Transcriptase (TIANGEN, China). The PCR reaction was performed on a STEP ONE PLUS Real-Time PCR System (Applied Biosystems, USA) following the manufacturer’s instructions. The 20 μl reactions were composed of 10 μl 2×RealMasterMix (TIANGEN, China), 1 μl forward primer, 1 μl reverse primers, 1μlcDNA template (diluted 100-fold with deionized water), and 7 μl sterile ddH_2_O. The PCR conditions were as follows: 94°C for 2 min, followed by 45 cycles of 94°C for 20 s, 60°C for 35 s and 68°C for 1 min. For all samples, three independent biological replicates were performed. Expression levels of the selected DEGs were calculated using the 2^-ΔΔCt^ method [83], with the 18S rRNA from *A*. *mongolicus* serving as the internal reference gene. The PCR primer information for this study is available in S2 Table.

## Supporting Information

S1 Fig GO classification of all PUTs. Based on Nr annotation, GO classification was performed, all PUTs were summarized into three main categories biological process, cellular component and molecular function.
(TIF)

S2 Fig Functional categories of stress-responsive DEGs. The drought-related DEGs were assigned to main functional categories based on the *Arabidopsis* MIPS classification scheme.
(TIF)

S3 Fig *A*. *mongolicus* under different levels of drought treatment. (a) *A*. *mongolicus* seedlings under 0, 2,3,4 weeks of drought treatment. (b) Expression levels of four drought related genes under 0, 2, 3, 4 weeks of drought treatment.
(JPG)

S1 Table The list of differentially expressed genes in *A*. *mongolicus*. (XLSX)

S2 Table The list of primers used in quantitative real-time PCR. (DOCX)

## References

[1] Zhu JK. Salt and drought stress signal transduction in plants. Annu Rev Plant Biol. 2002; 53: 247–273. 12221975 10.1146/annurev.arplant.53.091401.143329 PMC3128348

[2] Farooq M, Wahid A, Kobayashi N, Fujita D, Basra SMA. Plant drought stress: effects, mechanisms and management. Sust Agric. 2009; 29: 185–212.

[3] Yamaguchi-Shinozaki K, Shinozaki K. Transcriptional regulatory networks in cellular responses and tolerance to dehydration and cold stresses. Annu Rev Plant Biol. 2006; 57: 781–803. 16669782 10.1146/annurev.arplant.57.032905.105444

[4] Wang W, Vinocur B, Altman A. Plant responses to drought, salinity and extreme temperatures: towards genetic engineering for stress tolerance. Planta. 2003; 218: 1–14. 14513379 10.1007/s00425-003-1105-5

[5] Huang DQ, Wu WR, Abrams SR, Cutler AJ. The relationship of drought-related gene expression in *Arabidopsis thaliana* to hormonal and environmental factors. J Exp Bot. 2008; 59: 2991–3007. 10.1093/jxb/ern155 18552355 PMC2504347

[6] Qiu Q, Ma T, Hu Q, Liu B, Wu Y, Zhou H, et al Genome-scale transcriptome analysis of the desert poplar, *Populus euphratica* . Tree Physiol. 2011; 31: 452–461. 10.1093/treephys/tpr015 21427158

[7] Shi Y, Yan X, Zhao P, Yin H, Zhao X, Xiao H, et al Transcriptomic analysis of a tertiary relict plant, extreme xerophyte *Reaumuria soongorica* to identify genes related to drought adaptation. PloS One. 2013; 8: e63993 10.1371/journal.pone.0063993 23717523 PMC3662755

[8] Wang J, Wang Q, Yang Y, Liu X, Gu J, Li W, et al *De novo* assembly and characterization of stress transcriptome and regulatory networks under temperature, salt and hormone stresses in *Lilium lancifolium* . Mol Biol Rep. 2014; 41:8231–8245. 10.1007/s11033-014-3725-1 25200436

[9] Xie L, Yang Y. Miocene Origin of the Characteristic Broad-Leaved Evergreen Shrub *Ammopiptanthus* (Leguminosae) in the Desert Flora of Eastern Central Asia. Int J Plant Sci. 2012; 173: 944–955.

[10] Ge XJ, Yu Y, Yuan YM, Huang HW, Yan C. Genetic diversity and geographic differentiation in endangered *Ammopiptanthus* (Leguminosae) populations in desert regions of northwest China as revealed by ISSR analysis. Ann Bot. 2005; 95: 843–851. 15701663 10.1093/aob/mci089 PMC4246738

[11] Pang T, Ye CY, Xia X, Yin W. *De novo* sequencing and transcriptome analysis of the desert shrub, *Ammopiptanthus mongolicus*, during cold acclimation using Illumina/Solexa. BMC Genomics. 2013; 14: 488 10.1186/1471-2164-14-488 23865740 PMC3728141

[12] Chen JH, Sun Y, Sun F, Xia XL, Yin WL. Tobacco plants ectopically expressing the *Ammopiptanthus mongolicus AmCBL1* gene display enhanced tolerance to multiple abiotic stresses. Plant Growth Regul. 2011; 63: 259–269.

[13] Wei Q, Guo YJ, Cao HM, Kuai BK. Cloning and characterization of an AtNHX2-like Na+/H+ antiporter gene from *Ammopiptanthus mongolicus* (Leguminosae) and its ectopic expression enhanced drought and salt tolerance in *Arabidopsis thaliana* . Plant Cell Tiss Org. 2011; 105: 309–316.

[14] Wei Q, Hu P, Kuai BK. Ectopic expression of an *Ammopiptanthus mongolicus* H+-pyrophosphatase gene enhances drought and salt tolerance in *Arabidopsis* . Plant Cell Tiss Org. 2012; 110: 359–369.

[15] Cao P, Song J, Zhou C, Weng M, Liu J, Wang F, et al Characterization of multiple cold induced genes from *Ammopiptanthus mongolicus* and functional analyses of gene AmEBP1. Plant Mol Biol. 2009; 69: 529–539. 10.1007/s11103-008-9434-1 19067182

[16] Liu R, Liu M, Liu J, Chen Y, Chen Y, Lu C. Heterologous expression of a *Ammopiptanthus mongolicus* late embryogenesis abundant protein gene (*AmLEA*) enhances *Escherichia coli* viability under cold and heat stress. Plant Growth Regul. 2010; 60: 163–168.

[17] Wang Z, Gerstein M, Snyder M. RNA-Seq: a revolutionary tool for transcriptomics. Nat Rev Genet. 2009; 10: 57–63. 10.1038/nrg2484 19015660 PMC2949280

[18] Prentis PJ, Woolfit M, Thomas-Hall SR, Ortiz-Barrientos D, Pavasovic A, Lowe AJ, et al Massively parallel sequencing and analysis of expressed sequence tags in a successful invasive plant. Ann Bot. 2010; 106: 1009–1017. 10.1093/aob/mcq201 20929896 PMC2990670

[19] Xu D, Long H, Liang J, Zhang J, Chen X, Li JL, et al *De novo* assembly and characterization of the root transcriptome of *Aegilops variabilis* during an interaction with the cereal cyst nematode. Bmc Genomics. 2012; 13: 133 10.1186/1471-2164-13-133 22494814 PMC3439707

[20] Zhao Y, Thammannagowda S, Staton M, Tang S, Xia X, Yin W, et al An EST dataset for Metasequoia glyptostroboides buds: the first EST resource for molecular genomics studies in *Metasequoia* . Planta. 2013; 237: 755–770. 10.1007/s00425-012-1783-y 23117391

[21] Hiremath PJ, Farmer A, Cannon SB, Woodward J, Kudapa H, Tujita R, et al Large-scale transcriptome analysis in chickpea (*Cicer arietinum L*.), an orphan legume crop of the semi-arid tropics of Asia and Africa. Plant Biotechnol J. 2011; 9: 922–931. 10.1111/j.1467-7652.2011.00625.x 21615673 PMC3437486

[22] Novaes E, Drost DR, Farmerie WG, Pappas GJ, Grattapaglia D, Sederoff RR, et al High-throughput gene and SNP discovery in Eucalyptus grandis, an uncharacterized genome. BMC Genomics. 2008; 9: 312 10.1186/1471-2164-9-312 18590545 PMC2483731

[23] Hsiao YY, Chen YW, Huang SC, Pan ZJ, Fu CH, Chen WH, et al Gene discovery using next-generation pyrosequencing to develop ESTs for *Phalaenopsis orchids* . BMC Genomics. 2011; 12: 360 10.1186/1471-2164-12-360 21749684 PMC3146457

[24] Tang S, Liang H, Yan D, Zhao Y, Han X, Carlson JE, et al *Populus euphratica*: the transcriptomic response to drought stress. Plant Mol Biol. 2013; 83: 539–557. 10.1007/s11103-013-0107-3 23857471

[25] Liang HY, Ayyampalayam S, Wickett N, Barakat A, Xu Y, Landherr L, et al Generation of a large-scale genomic resource for functional and comparative genomics in *Liriodendron tulipifera* L. Tree Genet Genomes. 2011; 7: 941–954.

[26] Morozova O, Hirst M, Marra MA. Applications of New Sequencing Technologies for Transcriptome Analysis. Annu Rev Genom Hum Genet. 2009; 10: 135–151. 10.1146/annurev-genom-082908-145957 19715439

[27] Guo L, Wang D, Ma H, Yin W, Xia X. Complementary DNA-amplified fragment length polymorphism (AFLP-cDNA) analysis of differential gene expression from the xerophyte *Ammopiptanthus mongolicus* in response to cold, drought and cold together with drought. Afr J Biotechnol. 2011; 10: 3715–3727.

[28] Zhou Y, Gao F, Liu R, Feng J, Li H. *De novo* sequencing and analysis of root transcriptome using 454 pyrosequencing to discover putative genes associated with drought tolerance in *Ammopiptanthus mongolicus* . BMC Genomics. 2012; 13: 266 10.1186/1471-2164-13-266 22721448 PMC3407029

[29] Liu M, Shi J, Lu C. Identification of stress-responsive genes in *Ammopiptanthus mongolicus* using ESTs generated from cold- and drought-stressed seedlings. BMC Plant Biol. 2013; 13:88 10.1186/1471-2229-13-88 23734749 PMC3679971

[30] Wu Y, Wei W, Pang X, Wang X, Zhang H, Dong B, et al Comparative transcriptome profiling of a desert evergreen shrub, *Ammopiptanthus mongolicus*, in response to drought and cold stresses. BMC genomics. 2014; 15: 671 10.1186/1471-2164-15-671 25108399 PMC4143566

[31] Mortazavi A, Williams BA, Mccue K, Schaeffer L, Wold B. Mapping and quantifying mammalian transcriptomes by RNA-Seq. Nat Methods. 2008; 5: 621–628. 10.1038/nmeth.1226 18516045 PMC13303166

[32] Liu GH. Study on the endangered reasons of *Ammopiptanthus mongolicus* in the desert of Alashan. Bullet Botanical Res. 1998; 18: 341–345.

[33] Chaves MM, Maroco JP, Pereira JS. Understanding plant responses to drought—from genes to the whole plant. Funct Plant Biol. 2003; 30: 239–264. 10.1071/FP02076 32689007

[34] Schachtman DP, Goodger JQD. Chemical root to shoot signaling under drought. Trends Plant Sci. 2008; 13: 281–287. 10.1016/j.tplants.2008.04.003 18467158

[35] Atkinson NJ, Urwin PE. The interaction of plant biotic and abiotic stresses: from genes to the field. J Exp Bot. 2012; 63: 3523–3543. 10.1093/jxb/ers100 22467407

[36] Pandey GK, Cheong YH, Kim BG, Grant JJ, Li L, Luan S. CIPK9: a calcium sensor-interacting protein kinase required for low-potassium tolerance in *Arabidopsis* . Cell Res. 2007; 17: 411–421. 17486125 10.1038/cr.2007.39

[37] Ma S, Bohnert HJ. Integration of *Arabidopsis thaliana* stress-related transcript profiles, promoter structures, and cell-specific expression. Genome Biol. 2007; 8: R49 17408486 10.1186/gb-2007-8-4-r49 PMC1896000

[38] Mohammadi M, Kav NNV, Deyholos MK. Transcriptional profiling of hexaploid wheat (*Triticum aestivum* L.) roots identifies novel, dehydration‐responsive genes. Plant cell environ. 2007; 30: 630–645. 17407540 10.1111/j.1365-3040.2007.01645.x

[39] Todd J, Screen S, Crowley J, Peng J, Andersen S, Brown T, et al Identification and characterization of four distinct asparagine synthetase (AsnS) genes in maize (*Zea mays* L.). Plant Sci. 2008; 175: 799–808.

[40] Wang H, Liu D, Sun J, Zhang A. Asparagine synthetase gene TaASN1 from wheat is up-regulated by salt stress, osmotic stress and ABA. J Plant Physiol. 2005; 162: 81–89. 15700423 10.1016/j.jplph.2004.07.006

[41] Zhang XF, Jiang T, Wu Z, Du SY, Yu YT, Jiang SC, et al Cochaperonin CPN20 negatively regulates abscisic acid signaling in Arabidopsis. Plant Mol Biol. 2013; 83: 205–218. 10.1007/s11103-013-0082-8 23783410 PMC3777161

[42] Zhang J, Liu H, Sun J, Li B, Zhu Q, Chen S, et al Arabidopsis fatty acid desaturase FAD2 is required for salt tolerance during seed germination and early seedling growth. PLoS One. 2012; 7: e30355 10.1371/journal.pone.0030355 22279586 PMC3261201

[43] Gechev TS, Dinakar C, Benina M, Toneva V, Bartels D. Molecular mechanisms of desiccation tolerance in resurrection plants. Cell Mol Life Sci. 2012; 69: 3175–3186. 10.1007/s00018-012-1088-0 22833170 PMC11114980

[44] Szekely G, Abraham E, Cselo A, Rigo G, Zsigmond L, Csiszár J, et al Duplicated P5CS genes of *Arabidopsis* play distinct roles in stress regulation and developmental control of proline biosynthesis. Plant J. 2008; 53: 11–28. 17971042 10.1111/j.1365-313X.2007.03318.x

[45] Ruiz-Lozano JM, Porcel R, Aroca R. Evaluation of the possible participation of drought-induced genes in the enhanced tolerance of arbuscular mycorrhizal plants to water deficit. Mycorrhiza. 2008; 185–205.

[46] Alexandersson E, Danielson JAH, Rade J, Moparthi VK, Fontes M, Kjellbom P, et al Transcriptional regulation of aquaporins in accessions of *Arabidopsis* in response to drought stress. Plant J. 2010; 61: 650–660. 10.1111/j.1365-313X.2009.04087.x 19947979

[47] Bhushan D, Pandey A, Choudhary MK, Datta A, Chakraborty S, Chakraborty N. Comparative proteomics analysis of differentially expressed proteins in chickpea extracellular matrix during dehydration stress. Mol Cell Proteomics. 2007; 6(11): 1868–1884. 17686759 10.1074/mcp.M700015-MCP200

[48] Dani V, Simon WJ, Duranti M, Croy RR. Changes in the tobacco leaf apoplast proteome in response to salt stress. Proteomics. 2005; 5(3): 737–745. 15682462 10.1002/pmic.200401119

[49] Dinakar C, Djilianov D, Bartels D. Photosynthesis in desiccation tolerant plants: energy metabolism and antioxidative stress defense. Plant Sci. 2012; 182: 29–41. 10.1016/j.plantsci.2011.01.018 22118613

[50] Ali GM, Komatsu S. Proteomic analysis of rice leaf sheath during drought stress. J Proteome Res. 2006; 5: 396–403. 16457606 10.1021/pr050291g

[51] Bota J, Medrano H, Flexas J. Is photosynthesis limited by decreased Rubisco activity and RuBP content under progressive water stress? New phytol. 2004; 162: 671–681. 10.1111/j.1469-8137.2004.01056.x 33873761

[52] Li B, He L, Guo S, Li J, Yang Y, Yan B, et al Proteomics reveal cucumber Spd-responses under normal condition and salt stress. Plant Physiol Biochem. 2013; 67: 7–14. 10.1016/j.plaphy.2013.02.016 23524299

[53] Zhou G, Yang LT, Li YR, Zou CL, Huang LP, Qiu LH, et al Proteomic analysis of osmotic stress-responsive proteins in sugarcane leaves. Plant Mol Biol Rep. 2012; 30: 349–359.

[54] Askari H, Edqvist J, Hajheidari M, Kafi M, Salekdeh GH. Effects of salinity levels on proteome of *Suaeda aegyptiaca* leaves. Proteomics. 2006; 6: 2542–2554. 16612795 10.1002/pmic.200500328

[55] Kimura M, Yamamoto YY, Seki M, Sakurai T, Sato M, Abe T, et al Identification of *Arabidopsis* genes regulated by high light-stress using cDNA microarray. Photochem Photobiol. 2003; 77: 226–233. 12785063 10.1562/0031-8655(2003)077<0226:ioagrb>2.0.co;2

[56] Chaves MM, Flexas J, Pinheiro C. Photosynthesis under drought and salt stress: regulation mechanisms from whole plant to cell. Ann Bot. 2009; 103: 551–560. 10.1093/aob/mcn125 18662937 PMC2707345

[57] Cowan KJ, Storey KB. Mitogen-activated protein kinases: new signaling pathways functioning in cellular responses to environmental stress. J Exp Biol. 2003; 206: 1107–1115. 12604570 10.1242/jeb.00220

[58] Kiegerl S, Cardinale F, Siligan C, Gross A, Baudouin E, Liwosz A, et al SIMKK, a mitogen-activated protein kinase (MAPK) kinase, is a specific activator of the salt stress-induced MAPK, SIMK. Plant Cell. 2000; 12: 2247–2258. 11090222 10.1105/tpc.12.11.2247 PMC150171

[59] Zhang XT, Cheng TC, Wang GH, Yan YF, Xia QY. Cloning and evolutionary analysis of tobacco MAPK gene family. Mol Biol Rep. 2013; 40: 1407–1415. 10.1007/s11033-012-2184-9 23079708

[60] Liu YH, Offler CE, Ruan YL. Regulation of fruit and seed response to heat and drought by sugars as nutrients and signals. Front Plant Sci. 2013; 4: 282 10.3389/fpls.2013.00282 23914195 PMC3729977

[61] Robaglia C, Thomas M, Meyer C. Sensing nutrient and energy status by SnRK1 and TOR kinases. Curr Opin Plant Biol. 2012; 15: 301–307. 10.1016/j.pbi.2012.01.012 22305521

[62] Kulik A, Wawer I, Krzywińska E, Bucholc M, Dobrowolska G. SnRK2 protein kinases—key regulators of plant response to abiotic stresses. OMICS. 2011; 15: 859–872. 10.1089/omi.2011.0091 22136638 PMC3241737

[63] Zhang H, Mao X, Jing R. SnRK2 acts within an intricate network that links sucrose metabolic and stress signaling in wheat. Plant Signal Behav. 2011; 6: 652–654. 21448000 10.4161/psb.6.5.14945 PMC3172830

[64] Mao XG, Zhang HY, Tian SJ, Chang XP, Jing RL. TaSnRK2.4, an SNF1-type serine/threonine protein kinase of wheat (*Triticum aestivum* L.), confers enhanced multistress tolerance in *Arabidopsis* . J Exp Bot. 2010; 61: 683–696. 10.1093/jxb/erp331 20022921 PMC2814103

[65] Agarwal PK, Jha B. Transcription factors in plants and ABA dependent and independent abiotic stress signalling. Biol Plantarum. 2010; 54: 201–212.

[66] Uno Y, Furihata T, Abe H, Yoshida R, Shinozaki K, Yamaguchi-Shinozaki K, et al *Arabidopsis* basic leucine zipper transcription factors involved in an abscisic acid-dependent signal transduction pathway under drought and high-salinity conditions. Proc Nat Acad Sci. 2000; 97: 11632–11637. 11005831 10.1073/pnas.190309197 PMC17252

[67] Abe H, YamaguchiShinozaki K, Urao T, Iwasaki T, Hosokawa D, Shinozaki K. Role of *Arabidopsis* MYC and MYB homologs in drought- and abscisic acid-regulated gene expression. Plant Cell. 1997; 9: 1859–1868. 9368419 10.1105/tpc.9.10.1859 PMC157027

[68] Shinozaki K, Yamaguchi-Shinozaki K. Molecular responses to dehydration and low temperature: differences and cross-talk between two stress signaling pathways. Curr Opin Plant Biol. 2000; 3: 217–223. 10837265

[69] Liu Q, Kasuga M, Sakuma Y, Abe H, Miura S, Yamaguchi-Shinozaki K, et al Two transcription factors, DREB1 and DREB2, with an EREBP/AP2 DNA binding domain separate two cellular signal transduction pathways in drought- and low-temperature-responsive gene expression, respectively, in *Arabidopsis* . Plant Cell. 1998; 10: 1391–1406. 9707537 10.1105/tpc.10.8.1391 PMC144379

[70] Kizis D, Lumbreras V, Pages M. Role of AP2/EREBP transcription factors in gene regulation during abiotic stress. Febs Letters. 2001; 498: 187–189. 11412854 10.1016/s0014-5793(01)02460-7

[71] Shen YG, Zhang WK, He SJ, Zhang JS, Liu Q, Chen SY. An EREBP/AP2-type protein in Triticum aestivum was a DRE-binding transcription factor induced by cold, dehydration and ABA stress. Theor Appl Genet. 2003; 106: 923–930. 12647068 10.1007/s00122-002-1131-x

[72] Chen M, Wang QY, Cheng XG, Xu ZS, Li LC, Ye XJ, et al GmDREB2, a soybean DRE-binding transcription factor, conferred drought and high-salt tolerance in transgenic plants. Biochem Biophys Res Commun. 2007; 353: 299–305. 17178106 10.1016/j.bbrc.2006.12.027

[73] Zhou QY, Tian AG, Zou HF, Xie ZM, Lei G, Huang J, et al Soybean WRKY-type transcription factor genes,*GmWRKY13*, *GmWRKY21*, and *GmWRKY54*, confer differentialtolerance to abiotic stresses in transgenic *Arabidopsis* plants. Plant Biotechnol J. 2008; 6: 486–503. 10.1111/j.1467-7652.2008.00336.x 18384508

[74] Eulgem T. Dissecting the WRKY web of plant defenseregulators. PLOS Pathog. 2006; 2: e126 17121464 10.1371/journal.ppat.0020126 PMC1657070

[75] Luo X, Bai X, Sun X, Zhu D, Liu B, Ji W, et al Expression of wild soybean WRKY20 in Arabidopsis enhances drought tolerance and regulates ABA signalling. J Exp Bot. 2013; 64:2155–2169. 10.1093/jxb/ert073 23606412

[76] Levée V, Major I, Levasseur C, Tremblay L, MacKay J, Séguin A. Expression profiling and functional analysis of Populus WRKY23 reveals a regulatory role in defense. New Phytol. 2009; 184: 48–70. 10.1111/j.1469-8137.2009.02955.x 19674332

[77] Chang S, Puryear J, Cairney J. A simple and efficient method for isolating RNA from pine trees. Plant Mol Biol Rep. 1993; 11: 113–116.

[78] Gahlan P, Singh HR, Shankar R, Sharma N, Kumari A, Chawla V, et al *De novo* sequencing and characterization of *Picrorhiza kurrooa* transcriptome at two temperatures showed major transcriptome adjustments. BMC Genomics. 2012; 13: 126 10.1186/1471-2164-13-126 22462805 PMC3378455

[79] Conesa A, Gotz S, Garcia-Gomez JM, Terol J, Talon M, Robles M. Blast2GO: a universal tool for annotation, visualization and analysis in functional genomics research. Bioinformatics. 2005; 21: 3674–3676. 16081474 10.1093/bioinformatics/bti610

[80] Ye J, Fang L, Zheng HK, Zhang Y, Chen J, Zhang Z, et al WEGO: a web tool for plotting GO annotations. Nucleic Acids Res. 2006; 34: W293–W297. 16845012 10.1093/nar/gkl031 PMC1538768

[81] Kanehisa M, Araki M, Goto S, Hattori M, Hirakawa M, Itoh M, et al KEGG for linking genomes to life and the environment. Nucleic Acids Res. 2008; 36: D480–484. 18077471 10.1093/nar/gkm882 PMC2238879

[82] Iseli C, Jongeneel CV, Bucher P. ESTScan: a program for detecting, evaluating, and reconstructing potential coding regions in EST sequences. Proc Int Conf Intell Syst Mol Biol. 1999; 99: 138–148. 10786296

[83] Livak KJ, Schmittgen TD. Analysis of relative gene expression data using real-time quantitative PCR and the 2^−ΔΔCT^ method. Methods. 2001; 25: 402–408. 11846609 10.1006/meth.2001.1262

