# Correction: Characterization of the Transcriptome of the Xerophyte *Ammopiptanthus mongolicus* Leaves under Drought Stress by 454 Pyrosequencing

**DOI:** 10.1371/journal.pone.0140412

**Published:** 2015-10-08

**Authors:** The PLOS ONE Staff

The Funding statement is incorrect. The publisher apologizes for the error. The correct Funding statement is: This research was financially supported by the National Key Technologies R&D Program (2015BAD07B01), the National Natural Science Foundation of China (31270656, http://www.nsfc.gov.cn/), the Scientific Research and Graduate Training Joint Programs from BMEC and the Schatz Center for Tree Molecular Genetics Visiting Scholars Fund, Pennsylvania State University. The funders had no role in study design, data collection and analysis, decision to publish, or preparation of the manuscript.
