# Supplementary figures and images for: Characterization of the Transcriptome of the Xerophyte Ammopiptanthus mongolicus Leaves under Drought Stress by 454 Pyrosequencing
Source: PLoS One. 2015 Aug 27;10(8):e0136495. doi: 10.1371/journal.pone.0136495 (PMC4552034; doi:10.1371/journal.pone.0136495)

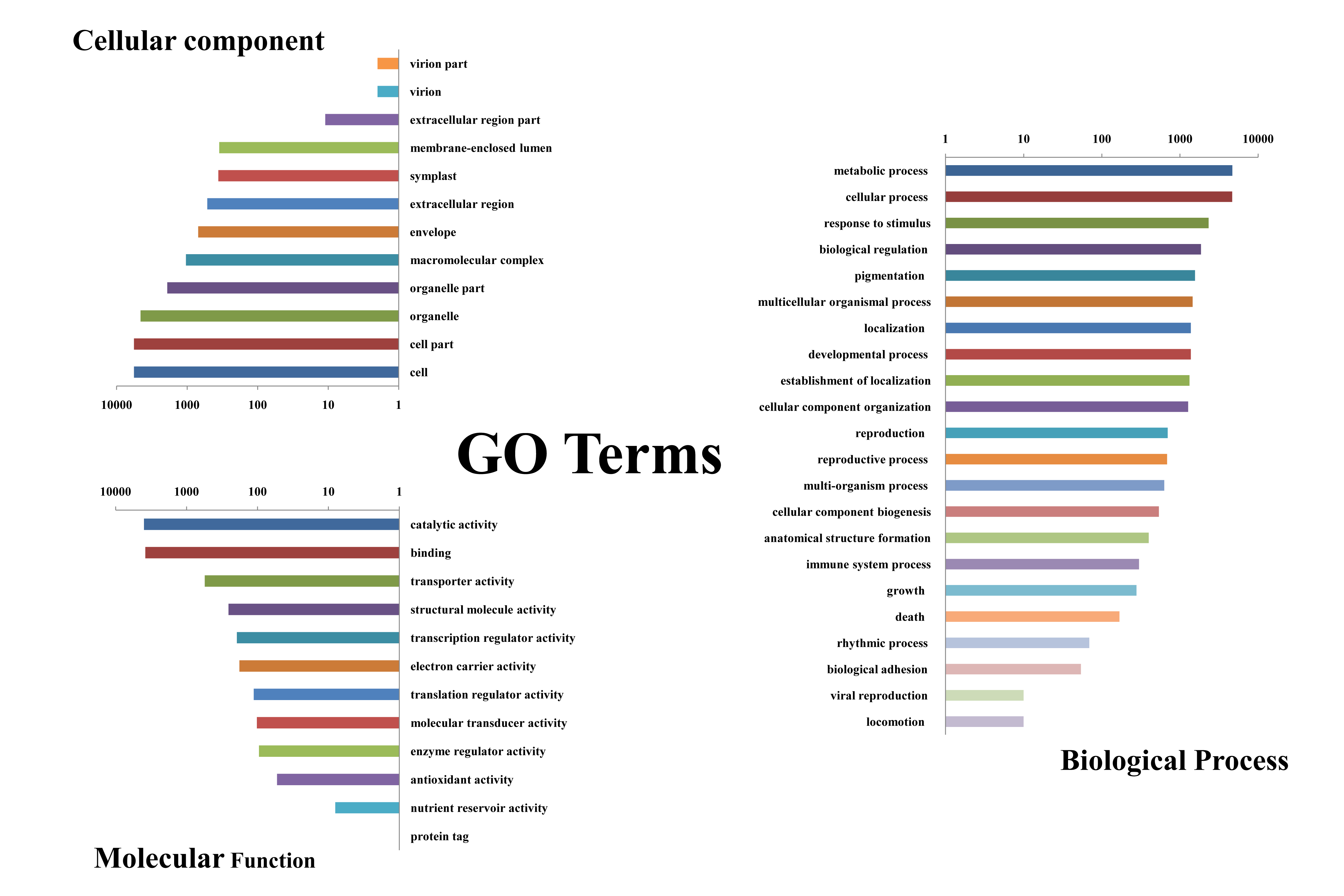

Supplement: S1 Fig — Based on Nr annotation, GO classification was performed, all PUTs were summarized into three main categories biological process, cellular component and molecular function. (TIF) [file pone.0136495.s001.tif]

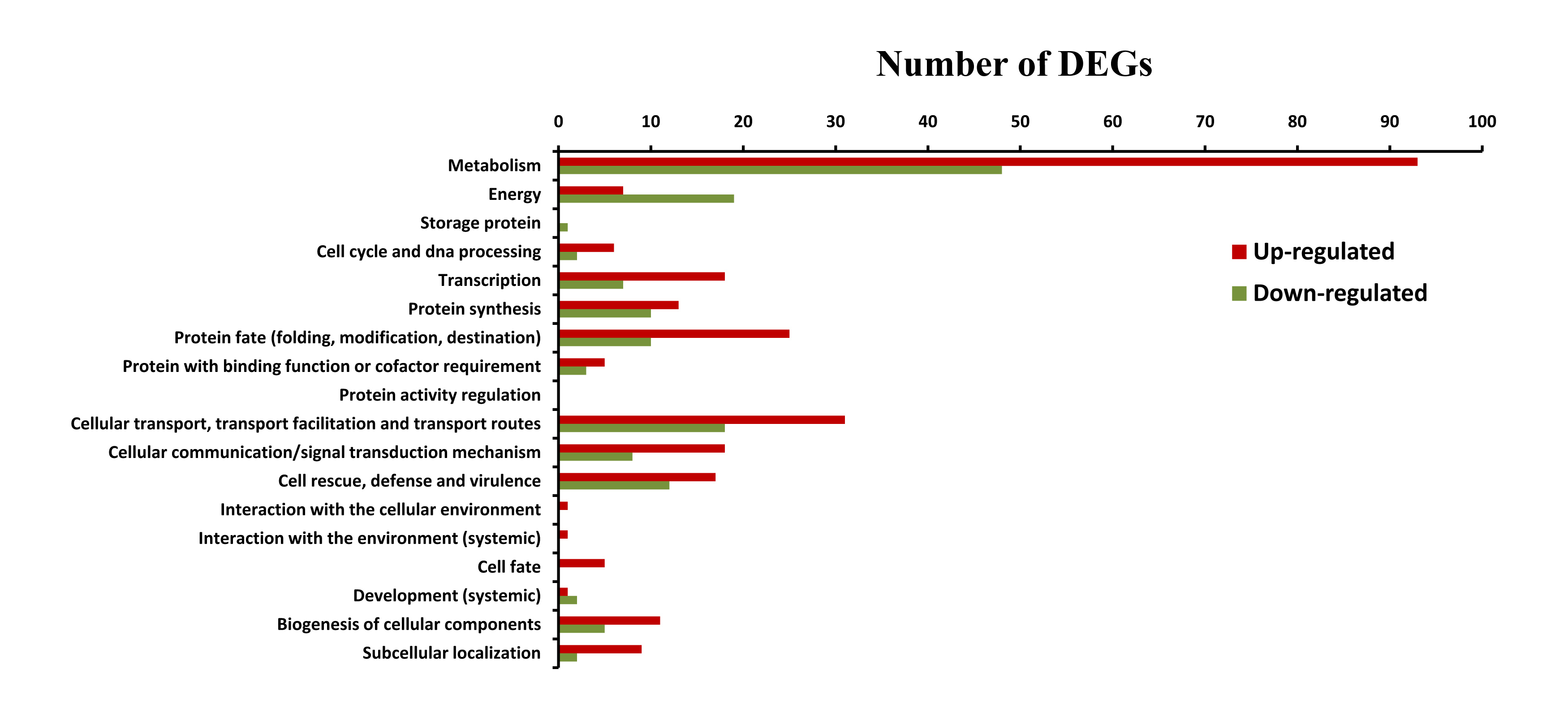

Supplement: S2 Fig — The drought-related DEGs were assigned to main functional categories based on the Arabidopsis MIPS classification scheme. (TIF) [file pone.0136495.s002.tif]

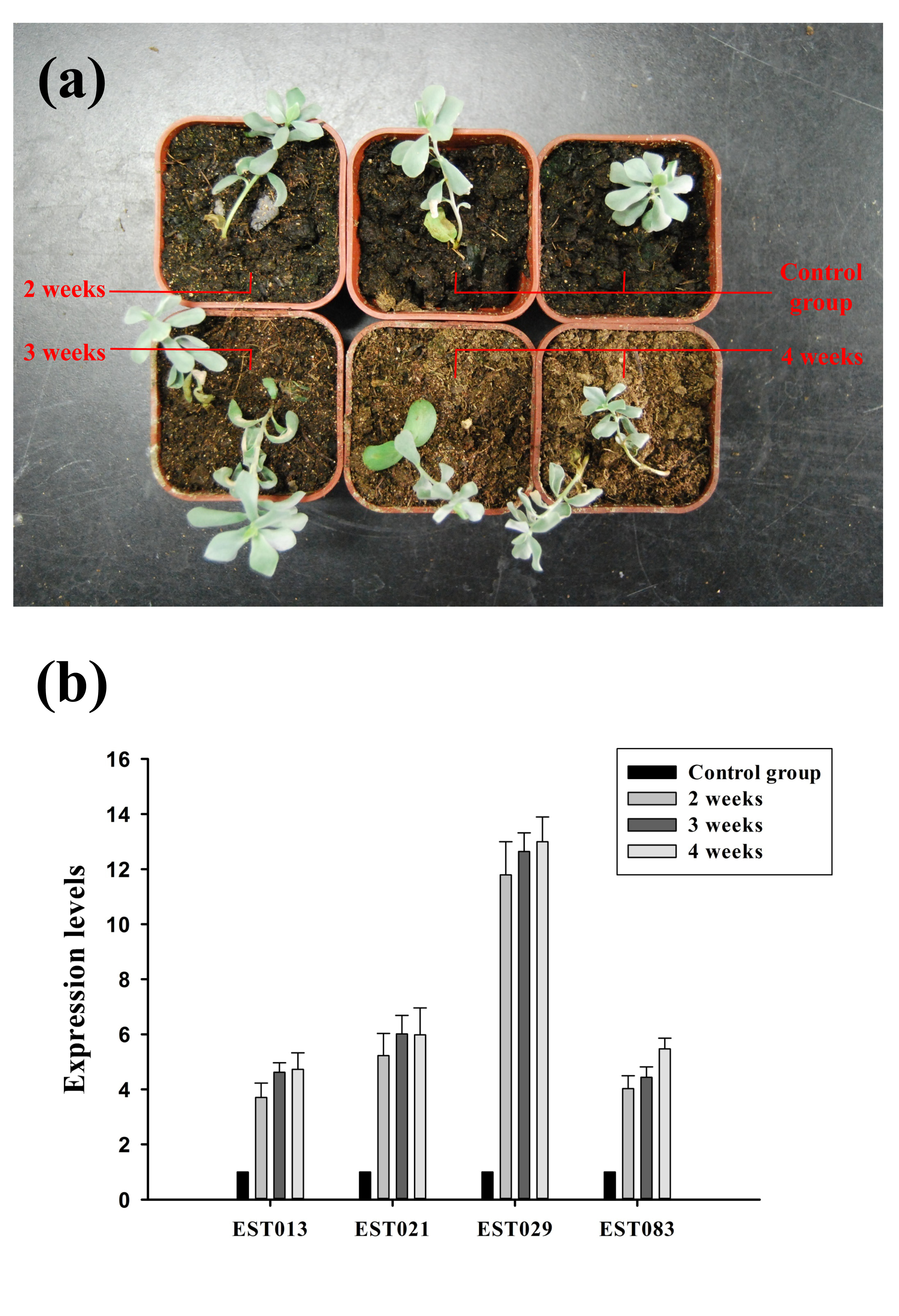

Supplement: S3 Fig — (a) A. mongolicus seedlings under 0, 2,3,4 weeks of drought treatment. (b) Expression levels of four drought related genes under 0, 2, 3, 4 weeks of drought treatment. (JPG) [file pone.0136495.s003.jpg]
