# Supplementary material for: Characterization of the Transcriptome of the Xerophyte Ammopiptanthus mongolicus Leaves under Drought Stress by 454 Pyrosequencing
Source: PLoS One. 2015 Aug 27;10(8):e0136495. doi: 10.1371/journal.pone.0136495 (PMC4552034; doi:10.1371/journal.pone.0136495)
Supplement: S2 Table — (DOCX) [file pone.0136495.s005.docx]

**Supplementary Material 2:** Primer sequences used for qRT-PCR

| **Gene** | **Forward primers (5’ to 3’)** | **Reverse primers (5’ to 3’)** |
| --- | --- | --- |
| CL231.Contig1 | TGCCTTAGCCAACCTTCTT | CCAATGACTCCTACGGTGC |
| Contig2563_DT | GGGATAGTTGGTGGTGTAAT | ACTTTGCGGAGACCTTGT |
| CL2.Contig2 | GAACACCAACACCAACACCA | CTGTGAGGCAAACTGACCAA |
| CL1037.Contig1 | ACAATTTGGGACCCATTC | GAGGATTTACGCCAGGAT |
| Contig3471_DT | GAACTCTGGCCACTGGATTC | CGACTTCGACGACCTTCTTC |
| CL73.Contig1 | CTCAAATTCGCAATACGC | AGTCCCTTCAGCTTCACC |
| Contig3094_DT | GATGGAAGTTGTGAAGGGTC | ATGGGAAGGAATGGAGGA |
| CL977.Contig1 | GGCCTCAACCTTTTTCCCTA | GTCAAACCTGGGTCAGAGGA |
| Contig1238_DT | GGTCCAGTAGCGTCCATC | CTAGTTTCCCAGTTACCAGAA |
| CL449.Contig1 | TAATCCCTACAAGGTATAGTTC | AGCAGTGTTGCCTCATAT |
| CL47.Contig1 | TAAAGAGGCTTAGAGGAGTT | TCTGGTTAGGAGGATTGC |
| CL372.Contig1 | AGGTGCTGGGATGCTTAG | GTGTAGGTGGCTCGTGGT |
| CL1086.Contig1 | CAACTTGAATCTTTCCTGCTT | AGCCCATCCATACCTTGC |
| Contig75_CK | GGGCAAACTTCATCTGGG | CTTGCACCTTGCATGGGT |
| CL79.Contig1 | AACGGATAGTTGTAACTTCG | ATGACCCATTCCTTTAGC |
| Contig3252_CK | CATGCTGCTTGTGCCTTTA | TCATCTGGTGGGCTGGTC |
| Contig831_CK | TGGGTCGGATTCAAGTGT | AAAGAAGTTGGCAGAGGAC |
| Contig598_CK | CTGGTGGAAGAGGAGATGA | GGTCAAAGGATAACGCAGT |
| Contig1027_CK | TCTGAGCGGCTGTATGAC | GCAGTTGGGAAGAACTTTA |
| CL1189.Contig1 | CTTTCCTTGACGCTGCTC | TGCCAACTGCCACTAACA |
| 18S | GGCTCTGCCCGTTGCTCT | CGTCACCCGTCACCACCA |
